# Miro GTPase domains regulate the assembly of the mitochondrial motor–adaptor complex

**DOI:** 10.26508/lsa.202201406

**Published:** 2022-10-27

**Authors:** Kayla Davis, Himanish Basu, Ismael Izquierdo-Villalba, Ethan Shurberg, Thomas L Schwarz

**Affiliations:** 1 F. M. Kirby Neurobiology Center, Boston Children’s Hospital, Boston, MA, USA; 2 Division of Medical Sciences, Harvard Medical School, Boston, MA, USA; 3 Department of Neurobiology, Harvard Medical School, Boston, MA, USA

## Abstract

Mitochondrial transport relies on a motor–adaptor complex containing Miro1, a mitochondrial outer membrane protein with two GTPase domains, and TRAK1/2, kinesin-1, and dynein. Using a peroxisome-directed Miro1, we quantified the ability of GTPase mutations to influence the peroxisomal recruitment of complex components. Miro1 whose N-GTPase is locked in the GDP state does not recruit TRAK1/2, kinesin, or P135 to peroxisomes, whereas the GTP state does. Similarly, the expression of the MiroGAP VopE dislodges TRAK1 from mitochondria. Miro1 C-GTPase mutations have little influence on complex recruitment. Although Miro2 is thought to support mitochondrial motility, peroxisome-directed Miro2 did not recruit the other complex components regardless of the state of its GTPase domains. Neurons expressing peroxisomal Miro1 with the GTP-state form of the N-GTPase had markedly increased peroxisomal transport to growth cones, whereas the GDP-state caused their retention in the soma. Thus, the N-GTPase domain of Miro1 is critical for regulating Miro1’s interaction with the other components of the motor–adaptor complex and thereby for regulating mitochondrial motility.

## Abstract (precis)

Relocalizing the mitochondrial motor–adaptor protein Miro to peroxisomes and systematically manipulating each GTPase domain reveal the importance of the N-terminal GTPase domain of Miro1 for regulating mitochondrial transport.

## Introduction

Mitochondria distribute themselves to ensure that they can respond to local metabolic demands and accomplish local calcium buffering and ROS signaling (Emptage et al, 2001; Mattson et al, 2008; Mochida et al, 2008; MacAskill et al, 2009a; Marchi et al, 2012). The proper distribution of mitochondria is critical for their inheritance during cell division and their contacts with other organelles (Kanfer et al, 2015; Chung et al, 2016; Modi et al, 2019). In animal cells, mitochondria are trafficked along microtubules by a motor–adaptor protein complex (Hirokawa et al, 1991; Stowers et al, 2002; Brickley et al, 2005; Glater et al, 2006; Pilling et al, 2006; van Spronsen et al, 2013). Disrupting this trafficking can lead to neurodegeneration (Hardy, 2010; Wang et al, 2011; Liu et al, 2012; Morotz et al, 2012; Nguyen et al, 2014; Zhang et al, 2015). The motor–adaptor complex includes Miro, a mitochondrial outer membrane protein with GTPase domains, TRAK, a motor–adaptor protein, and two microtubule-based motors (Stowers et al, 2002; Fransson et al, 2003, 2006; Glater et al, 2006; Brickley & Stephenson, 2011). The molecular motor kinesin-1 (KIF5) transports mitochondria toward the plus-end of microtubules, and the dynein-dynactin complex transports mitochondria toward their minus-ends (Vale et al, 1985; Hurd & Saxton, 1996; Tanaka et al, 1998; Chada & Hollenbeck, 2003; Glater et al, 2006; Pilling et al, 2006; Drerup et al, 2017). Additional components of the motor–adaptor complex have been found. Syntaphilin anchors mitochondria to microtubules, FHL2 anchors mitochondria to actin, and Myo19 drives short-range movements on actin filaments (Kang et al, 2008; Quintero et al, 2009; Chen & Sheng, 2013; Lopez-Domenech et al, 2018, 2021; Oeding et al, 2018; Seo et al, 2018; Bocanegra et al, 2020; Norkett et al, 2020; Basu et al, 2021). DISC1, O-GlcNAc transferase, and metaxins have also been implicated in the function and regulation of the motor–adaptor complex (Ogawa et al, 2014; Pekkurnaz et al, 2014; Zhao et al, 2021).

Mice and humans have two Miro genes, *RHOT1* and *RHOT2* (encoding Miro1 and Miro2), but their functional differences are not well understood (Fransson et al, 2003). Miro1 plays a central role in mitochondrial dynamics and is critical for mammalian development. Miro1 knockout is lethal in mice and decreases mitochondrial trafficking in somatic cells and neurons (Nguyen et al, 2014; Babic et al, 2015; Lopez-Domenech et al, 2016, 2018). Miro1 also participates in mitochondrial turnover, mitochondria-ER contact sites, and mitochondrial fission and fusion (Misko et al, 2010; Wang et al, 2011; Shlevkov et al, 2016; Lee et al, 2018; Lopez-Domenech et al, 2018, 2021; Modi et al, 2019; Bocanegra et al, 2020). There are four splicing variants of Miro1; some are known to localize to peroxisomes in at least some cell types or upon overexpression (Castro & Schrader, 2018; Okumoto et al, 2018; Covill-Cooke et al, 2020).

Miro has a C-terminal transmembrane domain that anchors the protein to mitochondria and two GTPase domains, one at the N-terminal and one near the C-terminal; these two GTPase domains are the focus of the current study. These GTPase domains are separated by two pairs of EF hands (Fransson et al, 2003; Klosowiak et al, 2013; Smith et al, 2020), which cause mitochondrial movement to stop when cytosolic Ca^2+^ increases (Saotome et al, 2008; Macaskill et al, 2009b; Wang & Schwarz, 2009). The functional significance of the GTPase domains has been harder to establish. Overexpressing Miro1 with its N-GTPase locked in a constitutively active GTP-bound state (P13V) alters mitochondrial distribution, whereas overexpressing mutations that place the N-GTPase in a constitutively inactive GDP-bound state (T18N) decreases mitochondrial trafficking and also alters their distribution (Saotome et al, 2008; Macaskill et al, 2009b; Wang & Schwarz, 2009). A *Vibrio cholerae*–derived protein (VopE) that activates the GTPase activity of Miro converts Miro to the GDP-bound state and thereby alters mitochondrial distribution (Suzuki et al, 2014). The T18N Miro1 mutation also prevents the binding of Myo19, CENP-F, and DISC1 (Kanfer et al, 2015; Oeding et al, 2018; Bocanegra et al, 2020; Norkett et al, 2020). Although these studies of the GTPase domains imply a regulatory role, the mechanism by which they alter mitochondrial distribution is less clear, particularly as concerns their transport by microtubule-based motors.

The use of overexpressed mutant forms to determine how the GTPase domains influence transport has been confounded by three issues: (1) the presence of the endogenous Miro1 and Miro2 on mitochondria, (2) the deleterious effects of grossly disturbing mitochondrial distribution and disrupting the other functions of Miro, and (3) the presence of other TRAK-binding proteins on mitochondria that may provide a parallel or alternative means of supporting transport (Misko et al, 2010; Lopez-Domenech et al, 2016). The formation of dimers between endogenous wild-type Miro and an expressed mutant, for example, can mask the consequences of the mutation on the assembly of the motor complex. The endogenous Miro or Miro-independent paths may also continue to mediate transport even when a completely inactive Miro is also expressed. To study the function of the GTPase domains with an independent approach and without these confounding factors, we have misdirected Miro transgenes to peroxisomes by using constructs that lack the mitochondria-targeting transmembrane domain and instead have a PEX3 peroxisome-targeting sequence. The peroxisome-targeted PEX3Miro1 construct is sufficient for the mislocalization of components of the motor–adaptor complex to peroxisomes. This strategy permitted us to compare the recruitment between motor–adaptor complex components to peroxisomes in the presence of mutations of both Miro1 GTPase domains. Because endogenous wild-type Miro was not detectable on these peroxisomes, their distribution in the cells was governed entirely by the expressed mutations. This system also allowed us to compare the functional differences between Miro1 and Miro2, and between TRAK1 and TRAK2, in a manner not possible on mitochondria.

## Results

### Mislocalization of Miro1 is sufficient for co-localization of the motor–adaptor complex to peroxisomes

To redirect Miro1 to peroxisomes, we used a construct in which the C-terminal transmembrane domain of Miro1 had been removed and the transmembrane domain of peroxisomal biogenesis factor, PEX3, had been added at the N-terminal of the construct (Basu et al, 2021). The N-terminal placement of the PEX3 transmembrane domain matches the orientation of the domain in PEX3 and localizes Miro1 to the peroxisomal surface. Between the N-terminus of Miro1 and the PEX3 transmembrane domain, the construct contained a 278–amino acid segment consisting of an mRFP tag, a 6×His tag, and an amino acid linker to create PEX3TM-6×His-mRFP-Miro1 (hereafter called PEX3Miro1). We also made a control construct lacking Miro1 (PEX3TM-6×His-mRFP, hereafter called PEX3-C).

**Figure 1.**
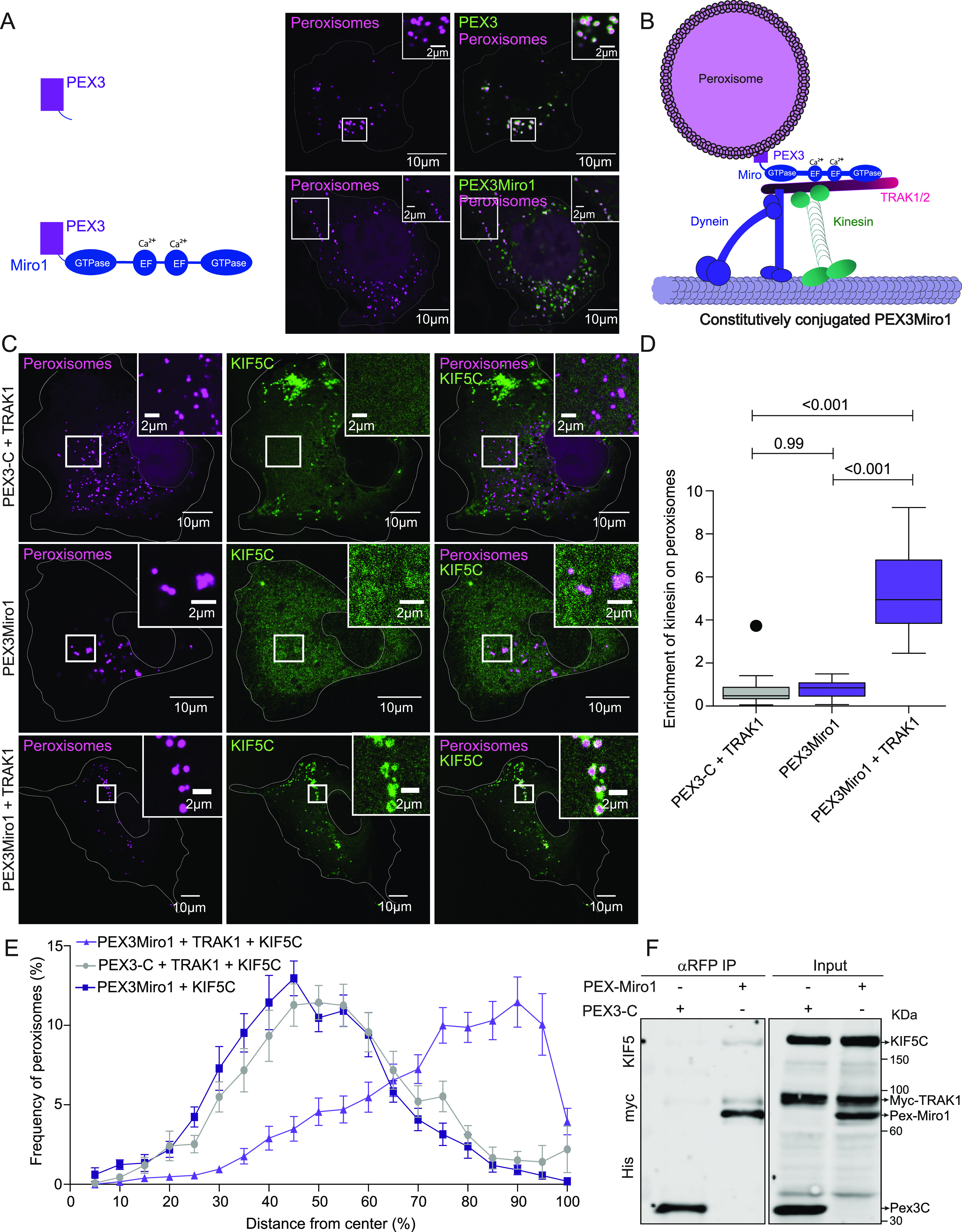
PEX3Miro1 localizes to peroxisomes and, with TRAK1 overexpression, can localize KIF5C to peroxisomes. **(A)** In COS-7 cells, expressed PEX3-6×His-mRFP (PEX3-C) or PEX3-6×His-mRFP-Miro1 (PEXMiro1) (green) co-localizes with the mTurquoise-SRL peroxisomal marker (magenta). Scale bar: 10 μm. Inset scale bar: 2 μm. **(B)** Schematic of the PEX3Miro1 construct recruiting TRAK1/2 and KIF5C to the surface of a peroxisome. **(C)** Expression of mTurquoise-SRL (magenta) with either PEX3-C or PEX3Miro1 and mCitrine-KIF5C (green) with or without the co-expression of myc-TRAK1 in COS-7 cells. The presence of PEX3-C and PEXMiro1 on peroxisomes was confirmed in each cell by imaging their RFP tags. Scale bar: 10 μm. Inset scale bar: 2 μm. **(D)** Quantification of the amount of KIF5C enriched on peroxisomes that carry PEX3-C control or PEX3Miro1 with or without the overexpression of myc-TRAK1. The quantifications here and subsequently are represented as “box-and-whisker” plots with the median value indicated. Outliers are represented as individual dots and are considered in statistical calculations. Here and in subsequent figures, *P*-values are indicated and were determined by one-way ANOVA with Dunnett’s T3 correction for multiple comparisons. **(E)** Distribution of peroxisomes marked with mTurquoise-SRL in the COS-7 cells analyzed in (D). Distance from the center of the cell in concentric shells was quantified using the DoveSonoPro FIJI macro (linked in the Materials and Methods section). N = 15 cells over three biological replicates. Error bars represent the SD. **(F)** PEX3-C or PEX3Miro1 was expressed in HEK293T cells along with myc-TRAK1 and mCitrine-KIF5C and co-immunoprecipitated using antibodies to the RFP tag on the PEX3-C and PEX3Miro1 constructs. Western blots were stained using anti-His, myc, and KIF5 antibodies. Data are from >3 biological replicates throughout. N = 15 for panels (A, C, D, E). The corner inserts show the enlargement of the boxed regions.

**Figure S1.**
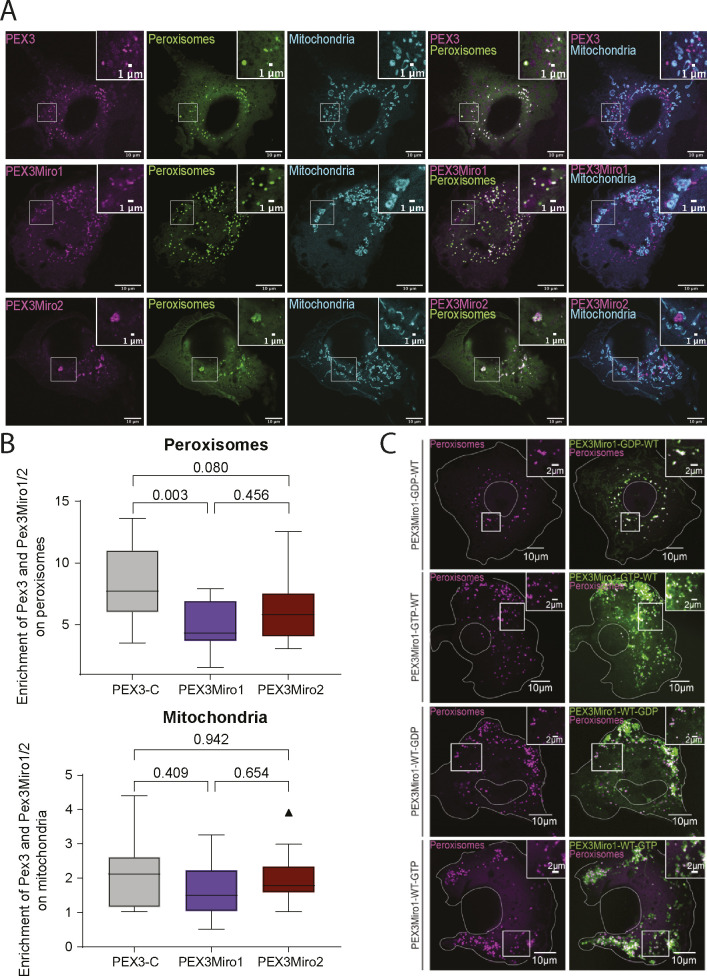
PEX3, PEX3Miro1, and PEX3Miro2 localization on peroxisomes and mitochondria. **(A)** COS-7 cells were co-transfected with PEX3-C, PEX3Miro1, or PEX3Miro2 together with the peroxisomal marker mCitrine-PTS1 (green) and the mitochondrial marker Mito-mTurquoise (blue). The corner inserts show the enlargement of the boxed regions. Scale bar: 10 μm. Inset scale bar: 1 μm. **(B)** From cells as in (A), the quantification of the RFP tags on PEX3-C and PEX3Miro that co-localized with the peroxisomal and mitochondrial markers. The data are represented as “box-and-whisker” plots with the median value indicated. The indicated *P*-values were obtained from one way-ANOVA with Dunnett’s T3 correction for multiple comparisons. N = 15 cells over three biological replicates. **(C)** PEX3Miro1 (green) localized appropriately to peroxisomes (magenta) despite mutations in the GTPase domain. The cells shown are the same as those in Fig 2A. The localization of KIF5C in Fig 2A is identical to that of PEX3Miro1 except in cells expressing PEX3Miro1 GDP-WT, where the KIF5C no longer co-localizes with the Miro construct and the peroxisomal marker.

Both PEX3Miro1 and PEX3-C were expressed in COS-7 cells with a peroxisomal marker, mTurquoise-serine-arginine-leucine (SRL), and appropriately localized to the outer membrane of peroxisomes (Figs 1A and S1A and B). When expressed at high levels, however, both constructs were also present on mitochondria, a consequence of the PEX3 transmembrane domain and not the Miro1 sequence (Fig S1B); mitochondrial localization upon overexpression can occur with full-length PEX3 as well (Sugiura et al, 2017). In subsequent experiments, we therefore restricted our analysis to signals that co-localized with the mTurquoise-SRL marker (unless otherwise noted) to avoid any confounding effects of spillover onto mitochondria (see the Materials and Methods section). In addition, to minimize variability in our observations due to differences in PEX3-C and PEX3Miro1 expression across cells and bioreplicates, transfection and imaging conditions were defined with an initial set of samples and kept constant throughout. Under these defined conditions, only cells having detectable amounts of PEX3Miro1 or PEX3-C co-localized with peroxisomes were considered for analysis.

To test whether the PEX3Miro1 construct was sufficient for relocalization of the motor–adaptor components to peroxisomes in COS-7 cells, we expressed either PEX3-C or PEX3Miro1 with mTurquoise-SRL and combinations of myc-TRAK1 and KIF5C, the neuron-specific isoform of KIF5, that was tagged with mCitrine and YFP (hereafter called mCitrine-KIF5C) (Fig 1B–D). After fixation, the extent of mCitrine-KIF5C co-localization with mTurquoise-SRL was quantified using a custom FIJI macro (see the Materials and Methods section). KIF5C was diffuse in the cytosol or present on mitochondria but absent from peroxisomes when co-expressed with PEX3-C and myc-TRAK1. Both in Fig 1C and in subsequent figures, whether mCitrine-KIF5C was diffuse in the cytosol or also detectable on mitochondria varied from cell to cell and was dependent on the expression level. In contrast, KIF5C was clearly present on peroxisomes when PEX3Miro1 and myc-TRAK1 were co-expressed (Fig 1C and D). Although variants of Miro1 have been reported to localize to peroxisomes (Costello et al, 2017; Okumoto et al, 2018; Covill-Cooke et al, 2020), there was a clear lack of detectable mCitrine-KIF5C on peroxisomes in the absence of PEX3Miro1. This lack of peroxisomal mCitrine-KIF5C indicates that if a peroxisome-targeted variant of Miro1 is endogenously present in these cells, it is not a significant factor and any KIF5C detected on peroxisomes in this and subsequent experiments is attributable to the PEX3Miro1 construct. These findings, and the ability to restrict our analysis to what is present on peroxisomes rather than on mitochondria, enable us to use the PEX3Miro system to study the regulation of the motor–adaptor complex without concern for the influence of endogenous mitochondrial Miro. When myc-TRAK1 was omitted, PEX3Miro1 was not sufficient to recruit visible amounts of mCitrine-KIF5C and did not significantly affect peroxisomal distribution (Fig 1C and D). This finding confirms the requirement for TRAK1 in the motor–adaptor complex (Stowers et al, 2002; Glater et al, 2006; van Spronsen et al, 2013; Henrichs et al, 2020), although we do not know why endogenous TRAK1 was not sufficient.

Peroxisomes in COS-7 cells typically reside near the nucleus. Although capable of microtubule-based transport (Rapp et al., 1996; Wiemer et al., 1997; Huber et al., 1999), peroxisomes in COS-7 cells moved little and were seldom encountered in the periphery of a cell. This perinuclear localization of peroxisomes was evident in transfections where the expressed mCitrine-KIF5C was not on peroxisomes (PEX3-C and TRAK1 or PEX3Miro1 without TRAK1). In contrast, when we expressed PEX3Miro1 with myc-TRAK1 and mCitrine-KIF5C, peroxisomes moved more (as in Basu et al [2021]) and were more widely dispersed in the cell (Fig 1E). We quantified their distribution using a laboratory-developed image analysis FIJI macro, DoveSonoPro (Basu et al, 2021). DoveSonoPro measures the percentage frequency of objects as they appear in concentric shells moving out from the center of the cell. The macro takes into consideration the shape of the cell and uses this shape to draw the concentric shells. Both the co-localization of KIF5C to peroxisomes and the increased peroxisomal dispersal were dependent on the co-expression of myc-TRAK1 with KIF5C (Fig 1E). As a further test of the ability of PEX3Miro1 to assemble the complex on peroxisomes, we expressed PEX3-C or PEX3Miro1 in HEK293T cells along with mCitrine-KIF5C and myc-TRAK1. myc-TRAK1 and mCitrine-KIF5C co-immunoprecipitated with PEX3Miro1 but not with the PEX3-C control (Fig 1F). These results establish that PEX3Miro1 is sufficient for localizing the proteins of the motor–adaptor complex and that the complex is active, that is, capable of moving peroxisomes in the direction of microtubule plus-ends, as expected for the KIF5C motor.

### The N-terminal GTPase determines Miro1 interactions with the mitochondrial motor–adaptor complex

**Figure 2.**
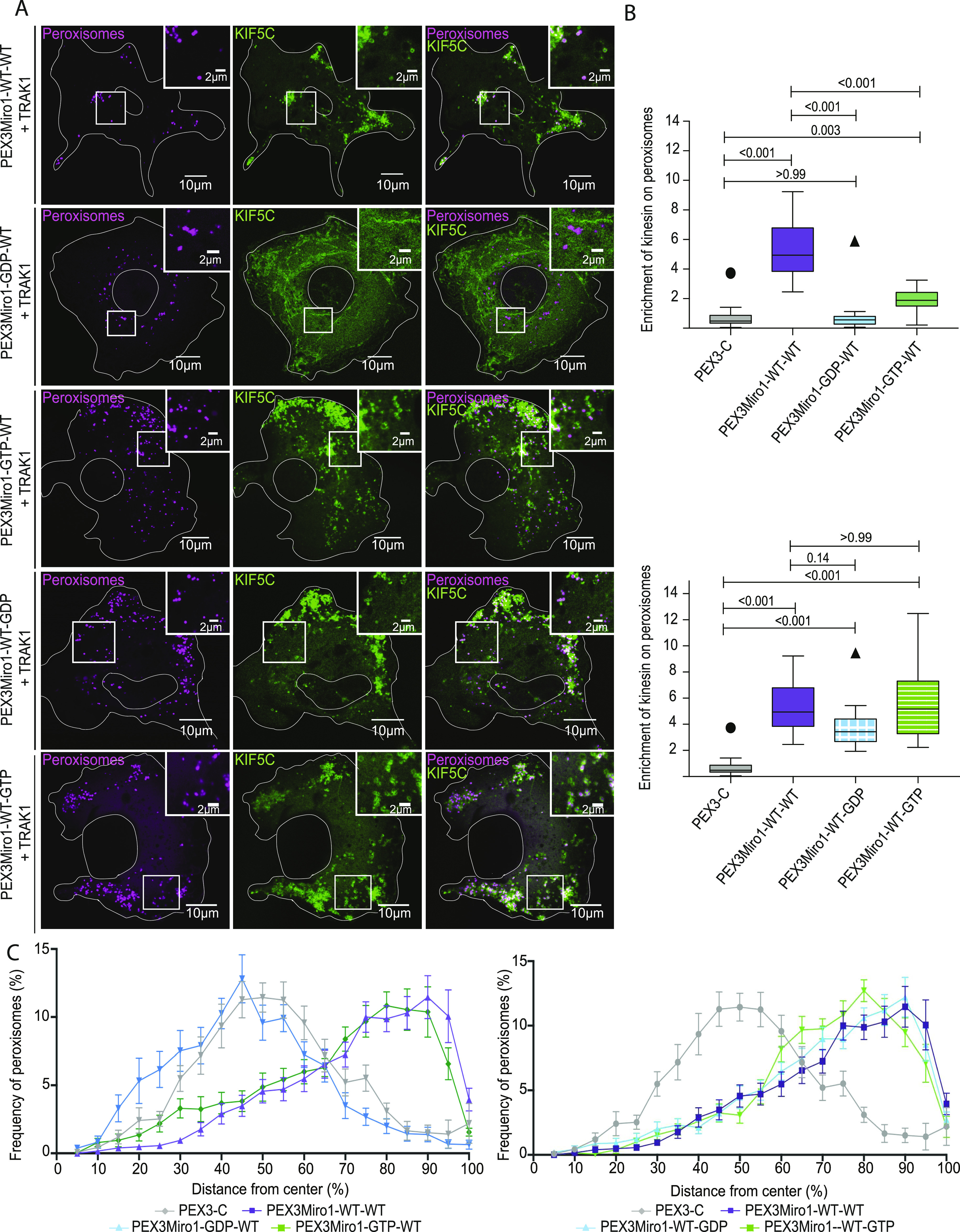
GTPase domains of Miro1 regulate co-localization with KIF5C on peroxisomes. **(A)** Expression in COS-7 cells of mTurquoise-SRL (magenta), myc-TRAK1, and mCitrine-KIF5C (green) with either PEX3Miro1 wild-type or PEX3Miro1 carrying a mutation of either the N- or C-GTPase domains: N-GDP-state T18N, N-GTP-state P13V, C-GDP-state S432N, and C-GTP-state K427N. The presence of the PEXMiro1 constructs on peroxisomes was confirmed in each cell by imaging their RFP tags. The corner inserts show the enlargement of the boxed regions. Here and in subsequent figures, the status of the two GTPase domains is abbreviated as either WT, GTP, or GDP for first the N-domain and then the C-domain. Scale bar: 10 μm. Inset scale bar: 2 μm. **(B)** Quantification of the amount of KIF5C enriched on peroxisomes carrying PEX3-C and the constructs shown in (A). For clarity, the mutations of the N-GTPase domain (above) are plotted separate from those of the C-GTPase (below) although transfected and imaged in the same experiments with the same controls. The quantification is represented as “box-and-whisker” plots with the median value indicated. Outliers are represented as single plot points and are included in all statistical calculations. *P*-values were determined by one-way ANOVA with Dunnett’s T3 correction for multiple comparisons. N = 15 cells over three biological replicates. **(C)** Quantification of peroxisomal distribution in concentric shells radiating from the center of the cell in the same cells analyzed in (B), with separate plots for mutations of the N-GTPase (left) and C-GTPase (right) for clarity, as in (B). N = 15 cells over three biological replicates. Error bars represent the SD. The data for the negative control (PEX3C) and the positive control (PEX3Miro1-WT-WT) are repeated from Fig 1 for clarity and are from experiments conducted at the same time.

The localization of a functional motor–adaptor complex to peroxisomes by PEX3Miro1 allowed us to examine the significance of GTPase mutations without confounding effects of endogenous Miro. We introduced single-point mutations to the GTPase domains of the PEX3Miro1 construct to confer either a constitutively active GTP state (N-GTPase:P13V or C-GTPase:K427N) or a constitutively inactive GDP state (N-GTPase:T18N or C-GTPase:S432N) (Fransson et al, 2006) (Fig 2). The constitutively active mutation is expected to lock the N-GTPase in a GTP-bound conformation by analogy to other GTPases (Smith et al, 2020). The inactive N-GTPase mutant locks the GTPase in a predicted GDP-bound conformation where GTP should no longer be able to bind.

To determine whether the N-GTPase domain regulates the assembly of the motor–adaptor complex, we quantified the effect of the PEX3Miro1 mutations on the localization of mCitrine-KIF5C to peroxisomes when co-expressed with TRAK1. The mRFP tag on these constructs confirmed that the mutated Miro constructs still localized to peroxisomes (Fig S1C), and thus, a failure to localize KIF5C to peroxisomes would reflect a failure of the complex to assemble on PEX3Miro1. When the N-GTPase carried the GDP-state T18N mutation, PEX3Miro1 no longer caused KIF5C to go to peroxisomes, whereas the GTP-state mutation (P13V) did (Fig 2A and B). Quantitatively, however, the GTP-state mutant did not recruit as much KIF5C as wild-type PEX3Miro1 (Fig 2B). This widely used constitutively active mutation thus may not be perfectly equivalent to wild-type PEX3Miro1 with a bound GTP; in addition to blocking the GTPase activity, it may slightly alter the structure of the domain and diminish its function. The cellular distribution of peroxisomes in these experiments was altered by successful KIF5C recruitment. Peroxisomes moved to the periphery in cells expressing wild-type or the GTP-state mutant of the N-GTPase of PEX3Miro1 but not in cells expressing the GDP-state mutant or the PEX3-C control (Fig 2C).

### Miro1 C-terminal GTPase mutations have little effect on KIF5C recruitment

Miro1 C-GTPase mutants have previously been overexpressed and altered mitochondrial distribution through an unknown mechanism (Fransson et al, 2006). We expressed PEX3Miro1 C-GTPase mutants in COS-7 cells together with mTurquoise-SRL, myc-TRAK1, and mCitrine-KIF5C and quantified KIF5C on peroxisomes. The mRFP tag on PEX3Miro1 confirmed that they were present on peroxisomes (Fig S1C). KIF5C was also present on peroxisomes in each of the C-GTPase mutants (Fig 2A and B). There may have been, however, subtle quantitative differences in peroxisomal KIF5C in the S432N C-GTPase GDP-state mutant (Fig 2B) relative to the wild-type or the K427N GTP-state mutant of PEX3Miro1 imaged in the same experiment. Consistent with the very small effects of C-GTPase mutations on KIF5C recruitment, they did not cause differences in the distribution of peroxisomes bearing them (Fig 2C).

**Figure 3.**
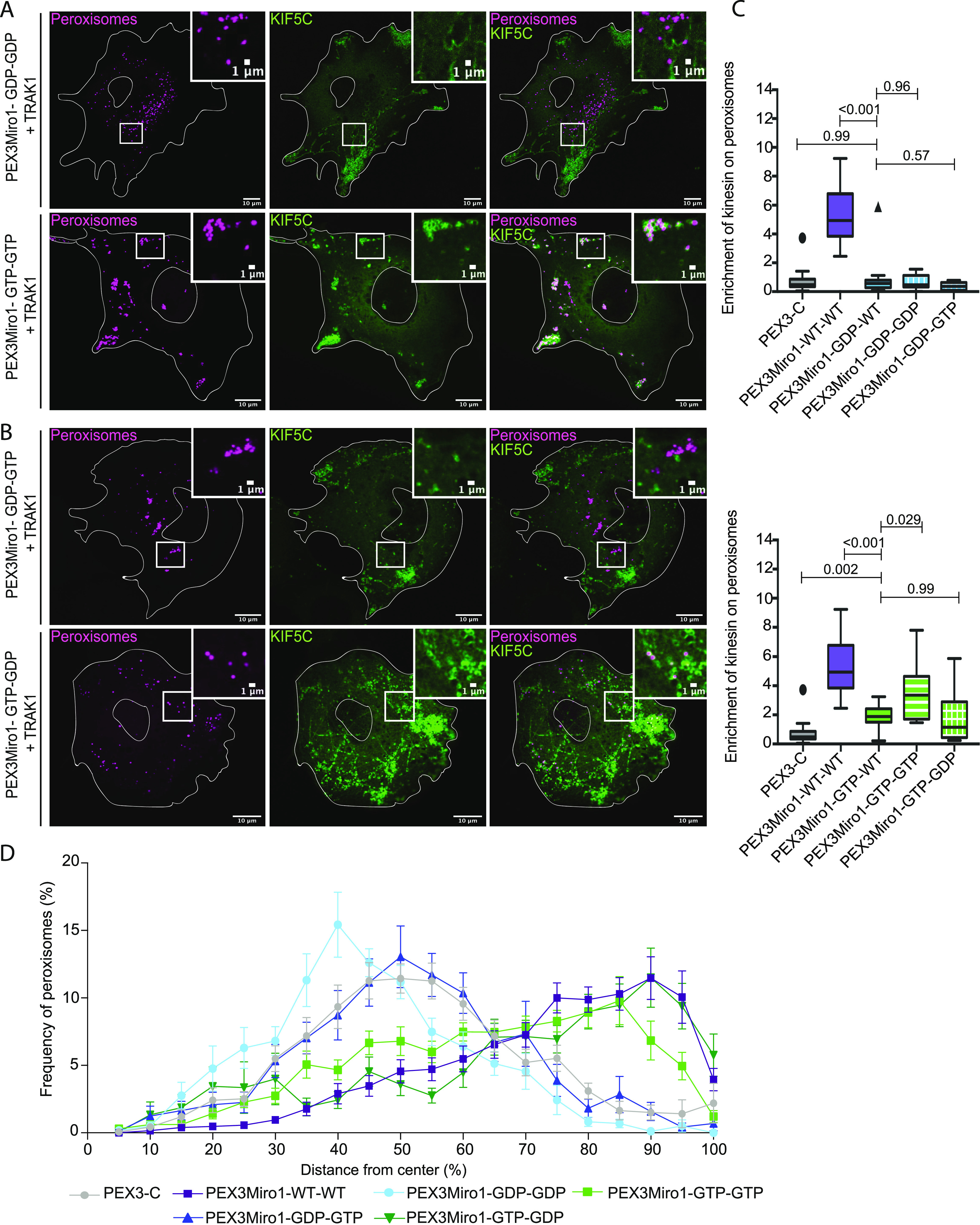
N-GTPase of Miro1 has a predominant influence on KIF5C recruitment. **(A)** Expression of PEX3Miro1 N-GTPase and C-GTPase double mutants in which both domains were in either the GTP or the GDP state and its consequences for mCitrine-KIF5C (green) recruitment to peroxisomes (mTurquoise-SRL, magenta) with the co-expression of myc-TRAK1. The corner inserts show the enlargement of the boxed regions. Scale bar: 10 μm. Inset scale bar: 1 μm. **(B)** Expression of PEX3Miro1 N-GTPase and C-GTPase double mutants in which the two domains were in opposite states and its consequences for mCitrine-KIF5C (green) recruitment to peroxisomes (mTurquoise-SRL, magenta) with the co-expression of myc-TRAK1. In (A, B), the presence of the PEXMiro1 constructs on peroxisomes was confirmed in each cell by imaging their RFP tags. **(C)** Quantification of the amount of KIF5C enriched on cells transfected as in (A, B). If the N-GTPase is in the GDP-bound state (upper graph) or in the GTP-bound state (lower graph), the state of the C-GTPase has little or no effect, except for a modest enhancement of KIF5C recruitment when both domains are GTP-bound rather than only the N-GTPase. The quantification is represented as “box-and-whisker” plots with the median value indicated. Outliers are represented as single plot points and are included in all statistical calculations. Indicated *P*-values are determined by one-way ANOVA with Dunnett’s T3 correction for multiple comparisons. N = 15 cells over three biological replicates. **(D)** Quantification of the peroxisomal distribution from the cells imaged for (C). Error bars represent the SD. The data for the negative control (PEX3C) and the positive control (PEX3Miro1-WT-WT) are repeated from Fig 1 for clarity and are from experiments conducted at the same time.

To further investigate a possible role for the PEX3Miro1 C-GTPase, we made PEX3Miro1 double mutants with both the N-GTPase and the C-GTPase in either the GTP state or the GDP state. Upon co-expression in COS-7 cells with mTurquoise-SRL, myc-TRAK1, and mCitrine-KIF5C, each double mutant matched the corresponding mutations of the N-GTPase alone. Peroxisomes with both domains in the GDP state did not co-localize with mCitrine-KIF5C, but those with both domains in the GTP state did (Fig 3A and C). We also made double mutants in which the two domains were locked in opposite states (Fig 3B and C). Again, the N-GTPase was the primary determinant of KIF5C recruitment; all the constructs in which the N-GTPase was in the GTP state recruited KIF5C to peroxisomes and shifted their distribution to the periphery, whereas all those in which the N-GTPase was in the GDP state did not (Fig 3D). Quantification, however, found that the construct with N-GTPase in the GTP state was slightly more effective in recruiting KIF5C to peroxisomes when the C-GTPase was in the GTP state than when it was in the WT or the GDP state (Fig 3C). Together, these results indicate that the state of PEX3Miro1 N-GTPase very largely determines the ability to recruit KIF5C.

### The Miro1 N-GTPase domain regulates the recruitment of P135

**Figure 4.**
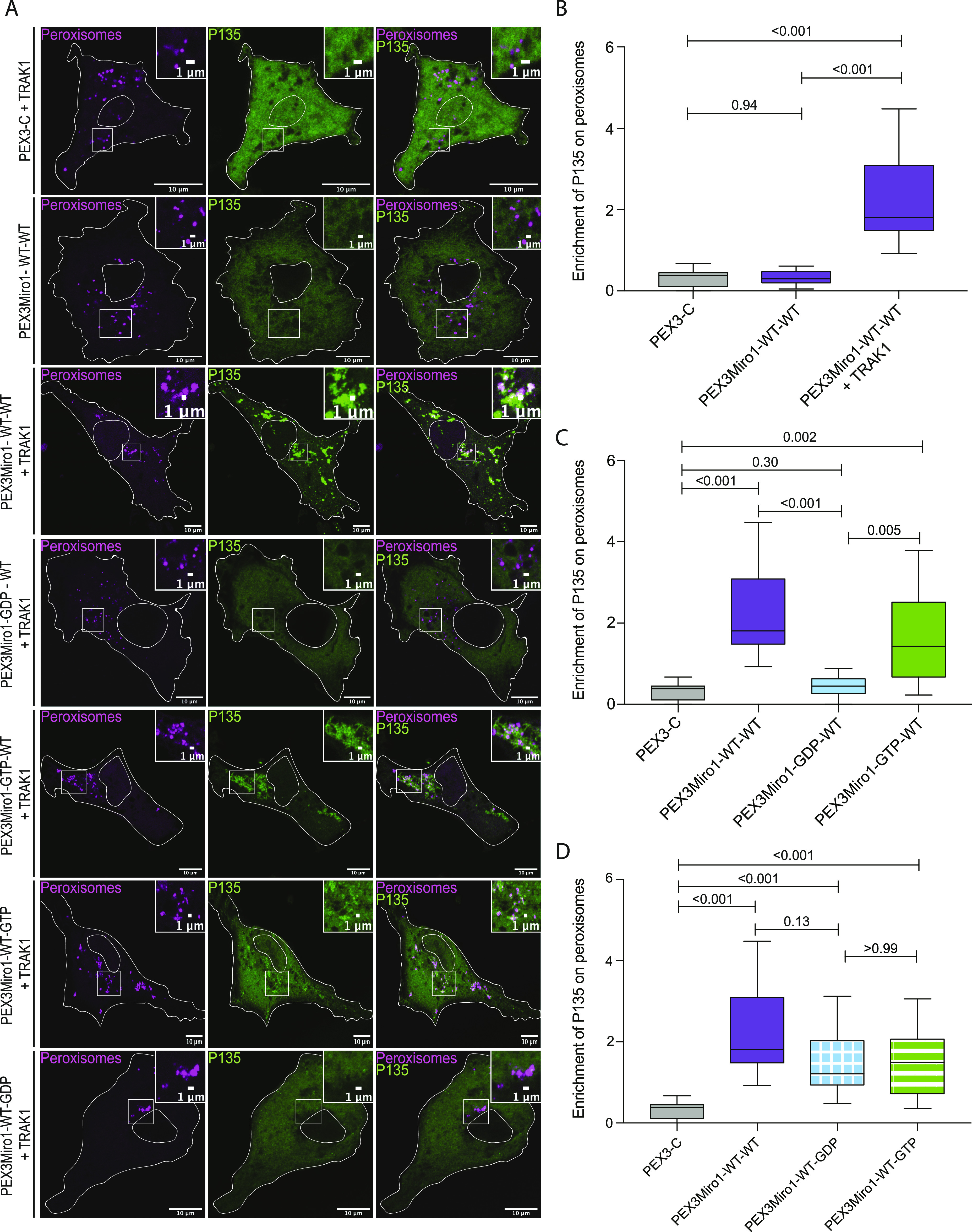
GTPase domains of Miro1 regulate co-localization with P135. **(A)** PEX3-C, PEX3Miro1, or PEX3Miro1 with mutations of either the N- or C-GTPase domains and with or without TRAK (as indicated) were expressed in COS-7 cells with mCitrine-P135 (green) and the peroxisomal marker mTurquoise-SRL (magenta). The presence of the PEXMiro1 constructs on peroxisomes was confirmed in each cell by imaging their RFP tags. The corner inserts show the enlargement of the boxed regions. Scale bar: 10 μm. Inset scale bar: 1 μm. **(B, C, D)** P135 co-localization with peroxisomes quantified from cells transfected as in (A) and represented as “box-and-whisker” plots with the median value indicated. Outliers are plotted as single points and are included in all statistical calculations. *P*-values were determined by one-way ANOVA with Dunnett’s T3 correction for multiple comparisons. N = 15 cells over three biological replicates. The data for the negative control (PEX3C) and the positive control (PEX3Miro1-WT-WT) are repeated in each graph for clarity and are from experiments conducted at the same time. **(B)** Quantification of the amount of P135 enriched on PEX3-C control or PEX3Miro1 peroxisomes with or without the expression of myc-TRAK1. **(C)** Quantification of the amount of P135 enriched on peroxisomes bearing PEX3Miro1 wild-type or the PEX3Miro1 N-GTPase mutants co-expressed with myc-TRAK1. **(D)** Quantification of the amount of P135 enriched on peroxisomes bearing PEX3Miro1 wild-type or the PEX3Miro1 C-GTPase mutants co-expressed with myc-TRAK1.

To examine the influence of the GTPase domains on the ability of the motor-adaptor complex to interact with the dynein-dynactin retrograde motor, we assayed the distribution of P150^Glued^, a microtubule-binding protein that is the largest subunit of dynactin. mCitrine-tagged P150^Glued^, however, when expressed in COS7 cells, coats all the microtubules, which made it impossible to determine if it was also present on PEX3Miro1-expressing peroxisomes. We therefore used mCitrine-tagged P135, a construct that lacks the microtubule-binding motif (Tokito et al, 1996; Dixit et al, 2008), and quantified P135 on peroxisomes tagged with mTurquoise-SRL. Like the KIF5C experiments, we found that PEX3Miro1 but not PEX3-C could recruit P135 to peroxisomes and that the overexpression of TRAK1 was necessary for this recruitment (Fig 4A and B). P135 recruitment was also assayed with GTPase mutations used in Figs 2 and 3. When the PEX3Miro1 N-GTPase was wild-type or carried the GTP-state mutation, P135 was recruited to peroxisomes. The N-GTPase GDP-state mutation did not recruit P135 (Fig 4A and C). The mutation of the C-GTPase to either the GTP or GDP state had less effect on P135 (Fig 4A and D). Thus, the regulation of P135 recruitment followed the same rules as KIF5C and was predominantly dependent on the state of the N-GTPase domain.

### The Miro1 N-GTPase regulates co-localization of TRAK1 and TRAK2 with PEX3Miro1

**Figure 5.**
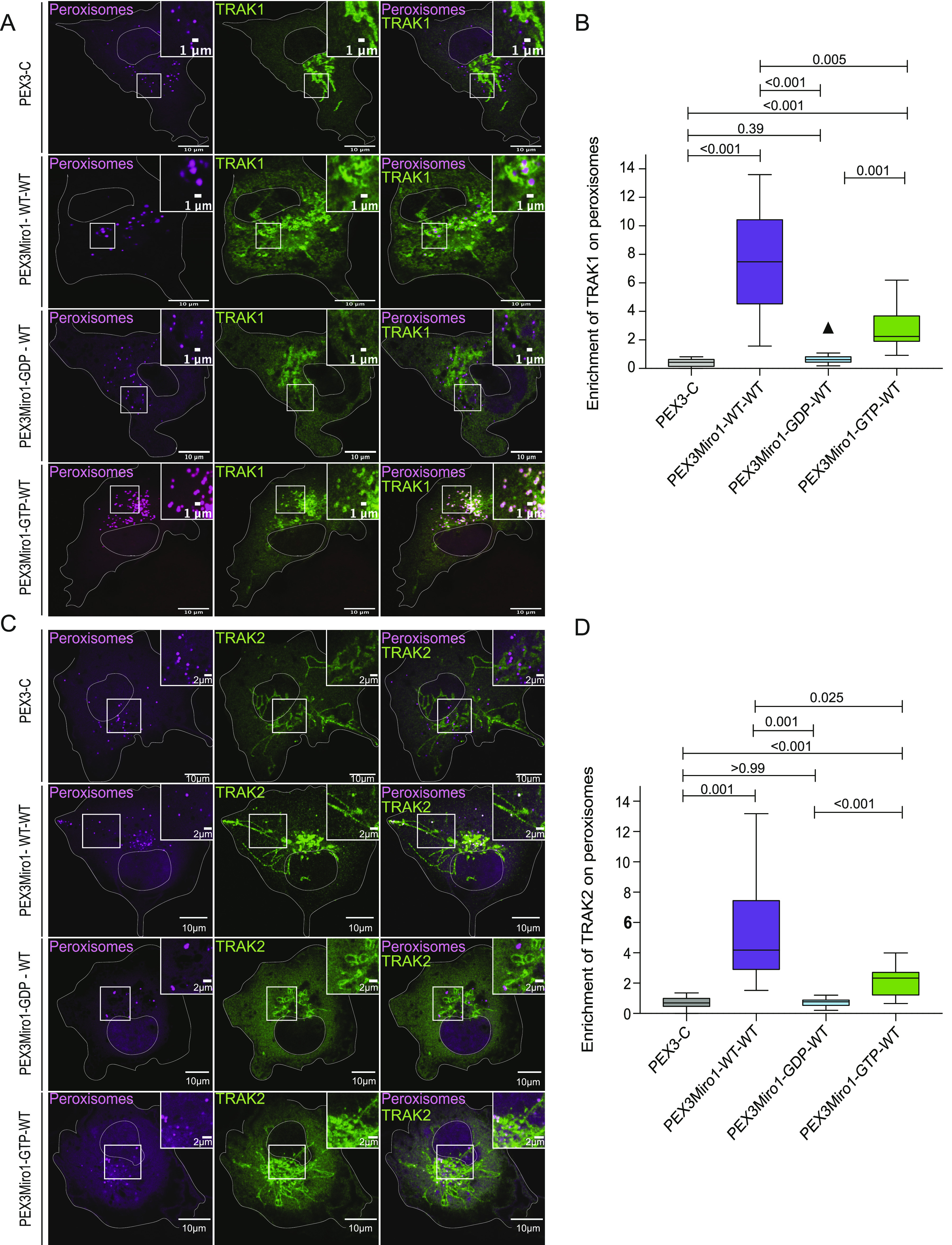
PEX3Miro1 N-GTPase regulates co-localization of TRAK1 and TRAK2 with PEX3Miro1. **(A)** PEX3-C, PEX3Miro1, or PEX3Miro1 with N-GTPase GDP- and GTP-state mutants was expressed in COS-7 cells with mCitrine-TRAK1 (green) and mTurquoise-SRL (magenta). The presence of the PEXMiro1 and PEX3-C constructs on peroxisomes was confirmed in each cell by imaging their RFP tags. Scale bar: 10 μm. Inset scale bar: 1 μm. **(B)** From cells as in (A), the quantification of the amount of TRAK1 enriched on peroxisomes bearing PEX3-C, PEX3Miro1, and PEX3Miro1 N-GTPase mutants. **(C)** PEX3-C, PEX3Miro1, or PEX3Miro1 with N-GTPase GDP- and GTP-state mutants was expressed in COS-7 cells with mCitrine-TRAK2 (green) and mTurquoise-SRL (magenta). The presence of the PEXMiro1 constructs on peroxisomes was confirmed in each cell by imaging their RFP tags. Scale bar: 10 μm. Inset scale bar: 2 μm. **(D)** From cells as in (C), the quantification of the amount of TRAK2 enriched on peroxisomes bearing PEX3-C, PEX3Miro1, and PEX3Miro1 N-GTPase mutants. In (B, D), TRAK co-localization with peroxisomes is represented as “box-and-whisker” plots with the median value indicated. Outliers are plotted as single points and are included in all statistical calculations. The indicated *P*-values are from analysis with one-way ANOVA. N = 15 cells over three biological replicates. N = 15 for all panels. The corner inserts show the enlargement of the boxed regions.

Consistent with most previous models of the motor-adaptor complex, our experiments indicated that TRAK was required for the association of the motors with Miro. We therefore hypothesized that the GDP state of the N-GTPase prevented the TRAK association with Miro and that the absence of TRAK on peroxisomes accounted for their failure to recruit KIF5C and P135. We co-expressed in COS7 cells the PEX3Miro1 N-GTPase mutants with mTurquoise-SRL and TRAK1 that was tagged with mCitrine and YFP (hereafter called mCitrine-TRAK1) and quantified the amount of mCitrine-TRAK1 on peroxisomes. Consistent with our hypothesis, both PEX3Miro1 N-GTPase wild-type and constitutively active mutants were able to recruit TRAK1 to peroxisomes, whereas the N-GTPase GDP-state mutant could not (Fig 5A and B).

**Figure S2.**
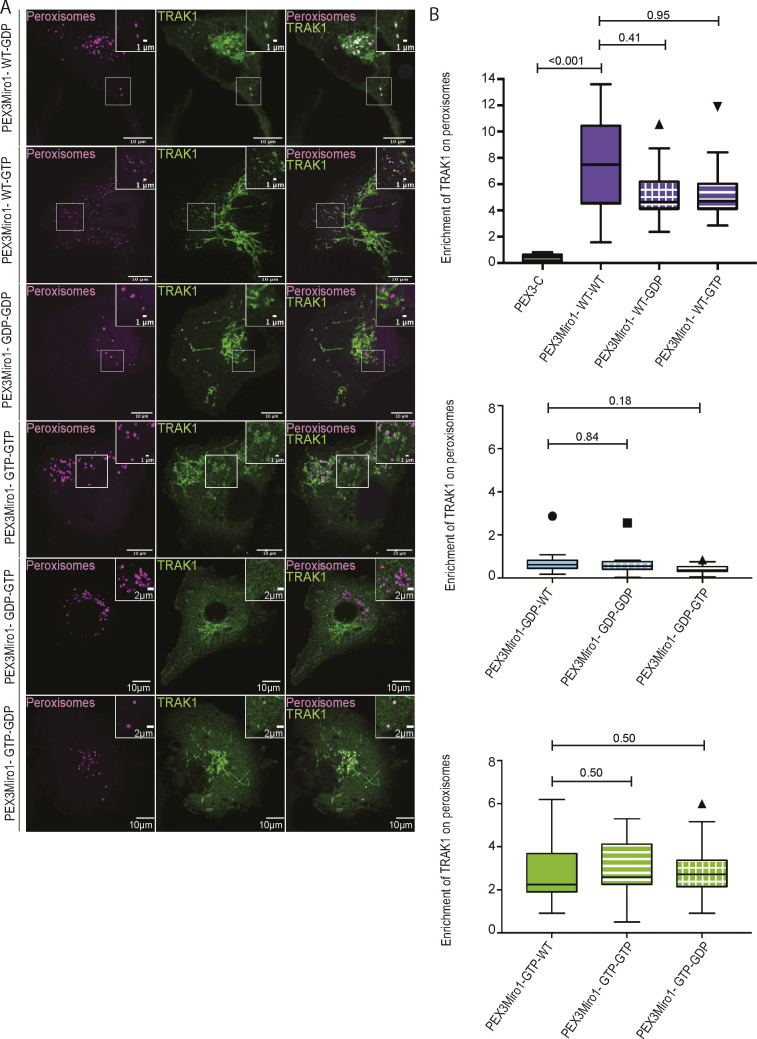
PEX3Miro1 C-GTPase has little influence on the co-localization of TRAK1 and PEX3Miro1. **(A)** PEX3Miro1, with the indicated mutations of the N- and C-GTPase domains, was co-expressed with mCitrine-TRAK1 (green) and mTurquoise-SRL (magenta) in COS-7 cells. The corner inserts show the enlargement of the boxed regions. Scale bar: 10 μm. Inset scale bar: marked as either 1 or 2 μm. (**B)** Quantification of the amount of TRAK1 enriched on peroxisomes transfected as in (A). The data are represented as “box-and-whisker” plots with the median value indicated. Outliers are represented as individual dots and are considered in all statistical calculations. The indicated *P*-values were determined by one-way ANOVA with Dunnett’s T3 correction for multiple comparisons. N = 15 cells over three biological replicates. All experiments were conducted and imaged in parallel except the GDP-GTP and GTP-GDP forms of PEXMiro1; they were performed together with controls that were consistent with the values of the other data shown.

**Figure S3.**
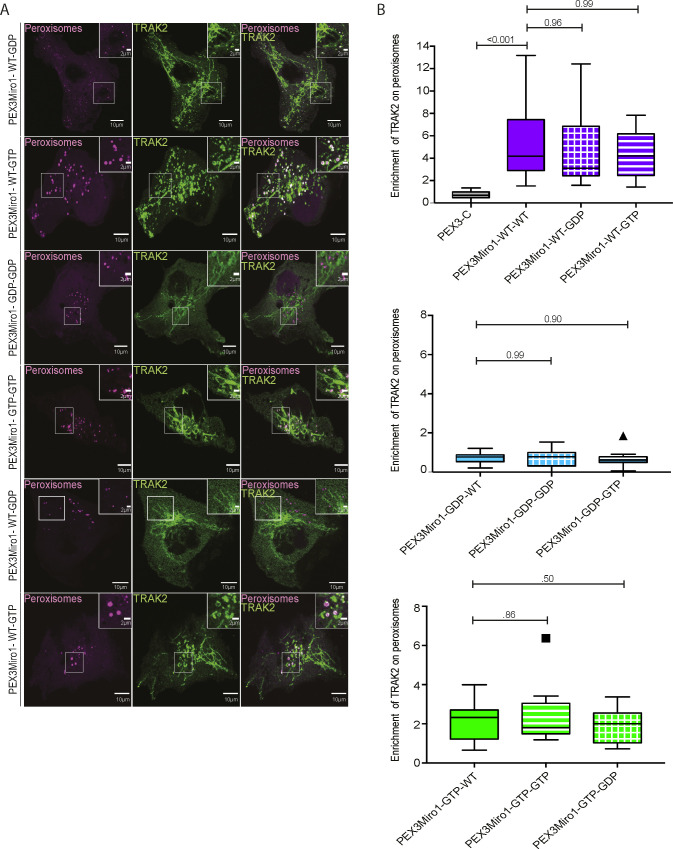
PEX3Miro1 C-GTPase has little influence on the co-localization of TRAK2 and PEX3Miro1. **(A)** PEX3Miro1, with the indicated mutations of the N- and C-GTPase domains, was co-expressed with mCitrine-TRAK2 (green) and mTurquoise-SRL (magenta) in COS-7 cells. The corner inserts show the enlargement of the boxed regions. Scale bar: 10 μm. Inset scale bar: 2 μm. **(B)** Quantification of the amount of TRAK2 enriched on peroxisomes transfected as in (A). The data are represented as “box-and-whisker” plots with the median value indicated. Outliers are represented as individual dots and are considered in all statistical calculations. The indicated *P*-values were determined by one-way ANOVA with Dunnett’s T3 correction for multiple comparisons. N = 15 cells over three biological replicates. All experiments were conducted and imaged in parallel except the GDP-GTP and GTP-GDP forms of PEXMiro1; they were performed together with controls that were consistent with the values of the other data shown.

Previous studies have indicated that the two TRAK proteins, TRAK1 and TRAK2, have functional differences (Brickley et al, 2005; Macaskill et al, 2009b; van Spronsen et al, 2013; Quintanilla et al, 2020). The PEXMiro assay allowed us to ask whether TRAK2 would also be recruited to PEXMiro1-expressing peroxisomes and be subject to the same regulation by the GTPase domains. We therefore co-expressed mCitrine-TRAK2 with PEX3Miro1 and mTurquoise-SRL. Like TRAK1, TRAK2 localized to peroxisomes with wild-type PEXMiro1 and PEXMiro1 with the N-GTPase in the GTP state, but not in the GDP state (Fig 5C and D). TRAK1 and TRAK2 also behaved similarly when the C-GTPase and double GTPase mutants were tested. Quantitatively, mCitrine-TRAK1 and mCitrine-TRAK2 on peroxisomes responded to the state of the Miro1 GTPase domains in the same way as mCitrine-KIF5C (Figs S2 and S3). We conclude that the N-GTPase of Miro1 controlled motor recruitment by determining whether or not Miro1 bound a TRAK.

**Figure 6.**
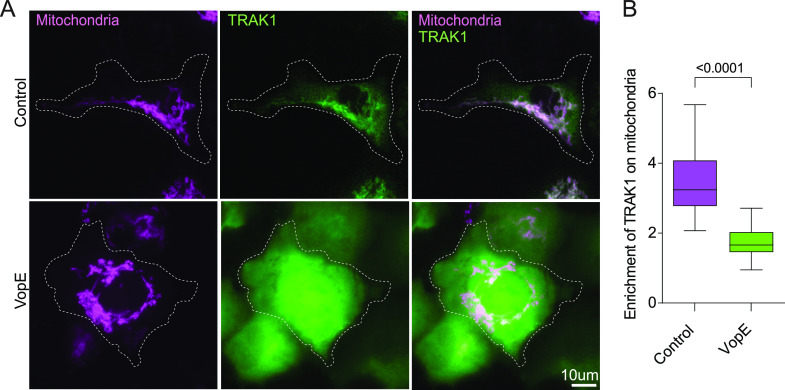
MiroGAP VopE causes TRAK1 to dissociate from mitochondria. **(A)** Representative images of COS-7 cells transfected with plasmids expressing mCitrine-TRAK1 (green) and mito-dsRED (magenta), and with or without a plasmid expressing VopE. Scale bar: 10 μm. **(B)** For each cell as in (A), the amount of TRAK1 present on mitochondria was quantified. The quantifications are represented as “Tukey’s box-and-whisker” plots with the median value indicated. The indicated *P*-value was derived from a two-tailed *t* test with Welsh’s correction. N = 100 cells from four independent experiments.

One caveat for this study and all previous studies of Miro’s GTPase domains is the reliance on mutations and the presumption that the T18N mutation exclusively alters the GTP/GDP state rather than directly interfering with binding TRAK or other Miro-binding proteins. To circumvent this concern, we took the independent approach of expressing VopE, a protein from *V. cholerae*. VopE is a Miro GTPase-activating protein (MiroGAP); that is, it binds to the GTPase domains of Miro and activates their GTPases with a resulting change in the distribution of mitochondria in *V. cholerae*–infected cells (Suzuki et al, 2014). In the absence of VopE, mCitrine-TRAK1 co-localized with mitochondria. Upon the co-expression of VopE, mCitrine-TRAK1 was released into the cytosol (Fig 6). This finding independently confirms the importance of the GTPase domains of Miro for governing TRAK recruitment and the assembly of the motor–adaptor complex.

### TRAK1 and TRAK2 differ in their ability to recruit KIF5C to peroxisomes

**Figure 7. Both:**
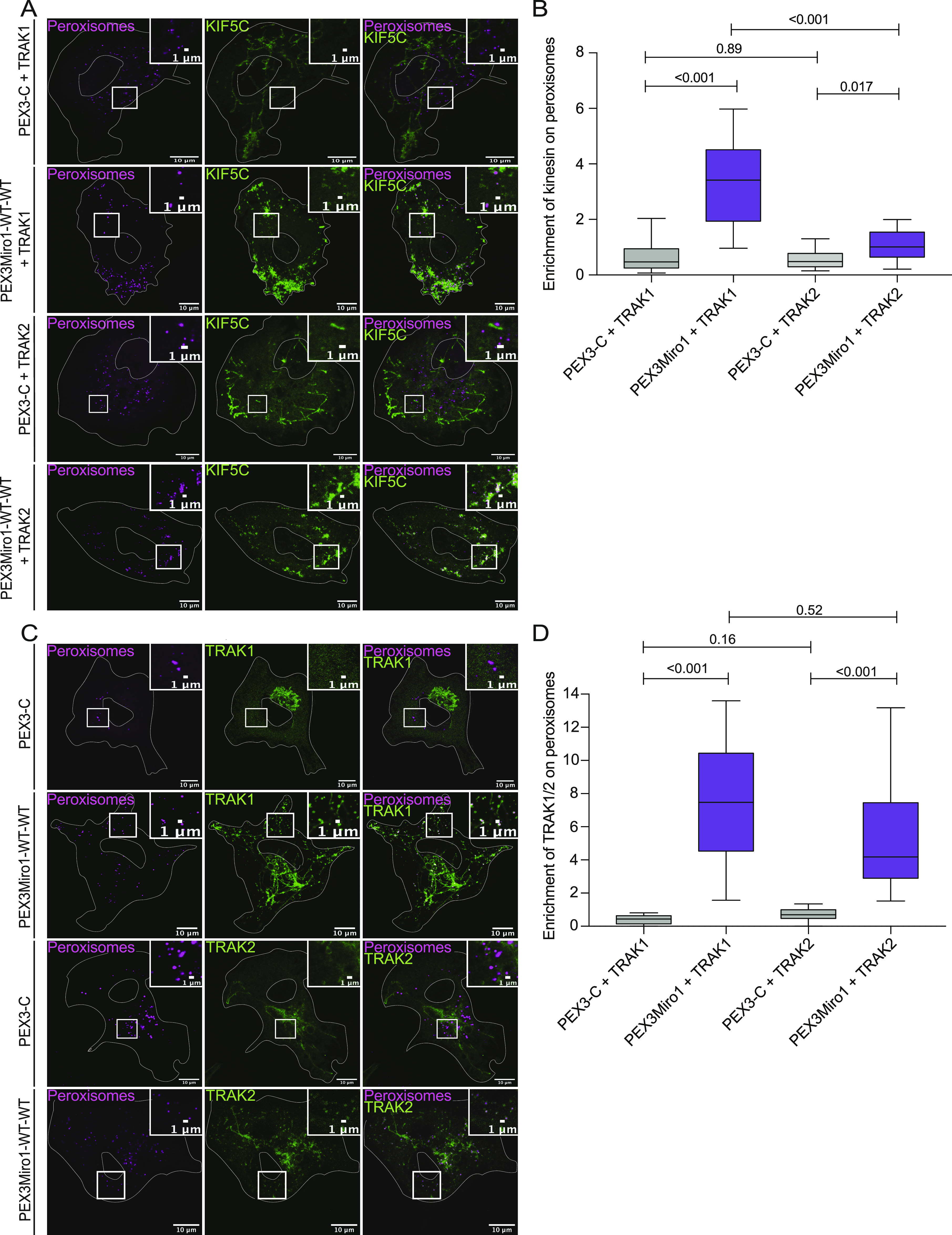
TRAK1 and TRAK2 serve as adaptors for KIF5C but differ in their ability to recruit KIF5C. **(A)** Either myc-TRAK1 or myc-TRAK2 was expressed in COS-7 cells together with mCitrine-KIF5C (green), mTurquoise-SRL (magenta), and PEX3-C or PEX3Miro1. The presence of the PEX3Miro1 or PEX3-C constructs on peroxisomes was confirmed in each cell by imaging their RFP tags. The corner inserts show the enlargement of the boxed regions. Scale bar: 10 μm. Inset scale bar: 1 μm. **(B)** From cells transfected as in (A), quantification of the amount of KIF5C enriched on peroxisomes with either myc-TRAK1 or myc-TRAK2. **(C)** Either mCitrine-TRAK1 or mCitrine-TRAK2 (green) was expressed in COS-7 cells together with mTurquoise-SRL (magenta) and PEX3-C or PEX3Miro1. The presence of the PEX3Miro1 and PEX3-C constructs on peroxisomes was confirmed in each cell by imaging their RFP tags. Scale bar: 10 μm. Inset scale bar: 1 μm. **(D)** From cells transfected as in (C), the quantification of the amount of mCitrine-TRAK1 or mCitrine-TRAK2 enriched on peroxisomes. In (B, D), co-localization with peroxisomes is represented as “box-and-whisker” plots with the median value indicated. The indicated *P*-values were determined by one-way ANOVA with Dunnett’s T3 correction for multiple comparisons. N = 15 cells over three biological replicates.

**Figure S4.**
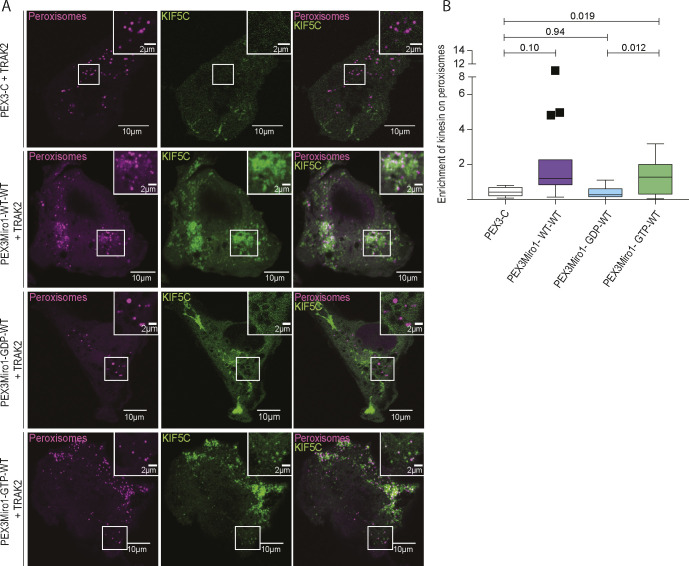
PEX3Miro1 N-GTPase regulates co-localization of KIF5C in the presence of TRAK2. **(A)** Either wild-type or N-GTPase mutations of PEX3Miro were expressed in COS-7 cells together with myc-TRAK2, mCitrine-KIF5C (green), and mTurquoise-SRL (magenta). Expression of PEX3Miro constructs on peroxisomes was confirmed by visualization of their RFP tags. The corner inserts show the enlargement of the boxed regions. Scale bar: 10 μm. Inset scale bar: 2 μm. **(B)** From cells as in (A), the quantification of mCitrine-KIF5C on peroxisomes. The data are represented as “box-and-whisker” plots with the median value indicated. The indicated *P*-values were determined by one-way ANOVA with Dunnett’s T3 correction for multiple comparisons. Outliers are represented as single plot points and are included in all statistical calculations. N = 15 cells over three biological replicates.

**Figure S5.**
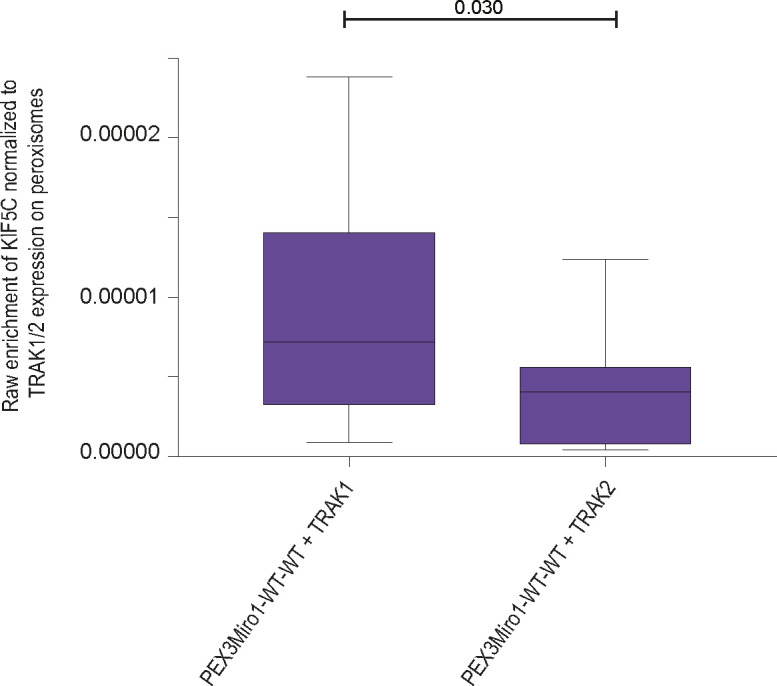
TRAK1 and TRAK2 differ in their recruitment of KIF5C to peroxisomes. COS-7 cells were transfected with mTurquoise-KIF5C, PEX3Miro1, and either mCitrine-TRAK1 or mCitrine-TRAK2. The amount of mTurquoise on peroxisomes was normalized to the intensity of mCitrine on peroxisomes to determine the relative efficacy of the two TRAKs isoforms for recruiting KIF5C. The data are represented as “box-and-whisker” plots with the median value indicated. The indicated *P*-value was obtained from the two-tailed unpaired *t* test with Welch’s correction. N = 15 cells over three biological replicates.

The requirement for overexpressed TRAK as an adaptor for the recruitment of KIF5C in the peroxisomal system allowed us to directly compare the function of TRAK1 with that of TRAK2 in serving as adaptors for KIF5C. We co-expressed PEX3Miro1 with mTurquoise-SRL, mCitrine-KIF5C, and either myc-TRAK1 or myc-TRAK2. Similar to TRAK1, TRAK2 expression could also localize KIF5C to peroxisomes (Fig 7), with the same dependency on the state of the N-GTPase domain (Fig S4). Comparatively, however, TRAK2 recruited significantly less KIF5C than TRAK1 (Fig 7A and B). This difference was not due to different amounts of TRAK1 and TRAK2 on peroxisomes; the co-expression of either mCitrine-TRAK1 or mCitrine-TRAK2 with PEX3Miro1 resulted in similar amounts of TRAK on peroxisomes (Fig 7C and D). We also quantified the ratio of peroxisomal KIF5C to peroxisomal TRAK1 and TRAK2 by co-expressing PEX3Miro1 with an mTurquoise-KIF5C construct in the presence of either mCitrine-TRAK1 or mCitrine-TRAK2. We normalized the amount of KIF5C to the amount of each TRAK and again found that more KIF5C co-localized with TRAK1 than with TRAK2 (Fig S5). Thus, in a comparison of the two TRAK isoforms, the difference in KIF5C recruitment to peroxisomes was dependent on differences in TRAK-KIF5C interactions and not differences in TRAK expression or TRAK-Miro interactions.

### PEX3Miro2 does not recruit the motor–adaptor complex to peroxisomes

**Figure S6.**
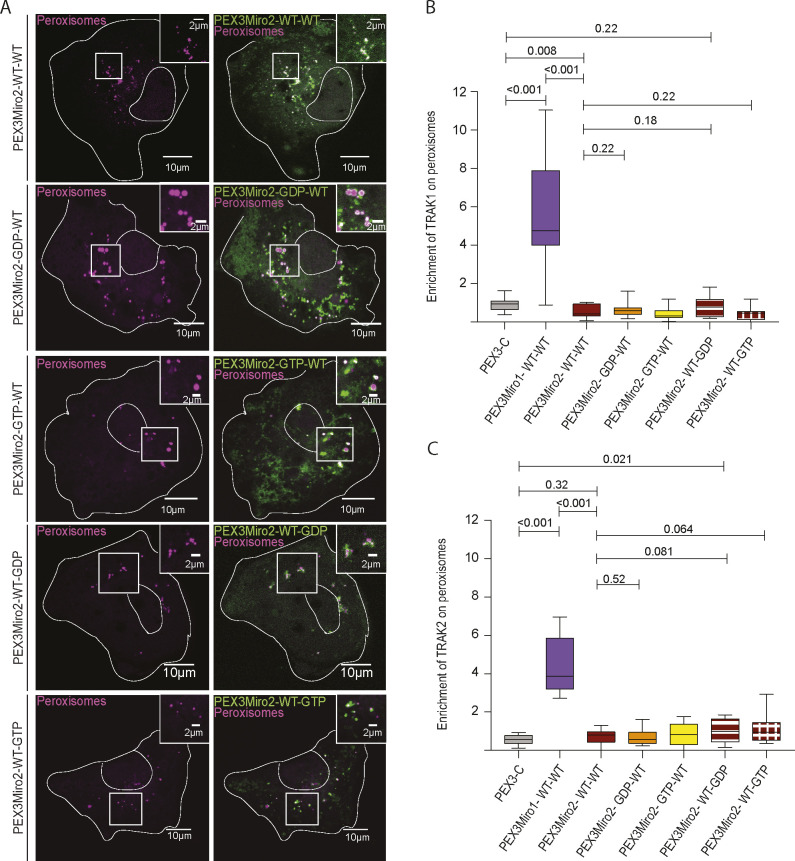
PEX3Miro2 does not recruit TRAK1 or TRAK2 to peroxisomes. **(A)** PEX3Miro2 (green) localized appropriately to peroxisomes (magenta). The cells shown are the same as those expressing PEX3Miro2 in Fig 8A. Scale bar: 10 μm. Inset scale bar: 2 μm. **(B, C)** Quantification of the amount of mCitrine-TRAK1 (B) or mCitrine-TRAK2 (C) enriched on peroxisomes carrying PEX3-C, PEX3Miro1, PEX3Miro2, or the PEX3Miro2 GTPase mutant constructs as indicated on the x-axis. Peroxisomes were marked with mTurquoise-SRL. The quantification is represented as “box-and-whisker” plots with the median value indicated. Outliers have been removed from this data set using the ROUT method (Q = 1%) and are not included in statistical calculations. Indicated *P*-values were determined by one-way ANOVA with Dunnett’s T3 correction for multiple comparisons. N = 15 cells over three biological replicates.

**Figure 8.**
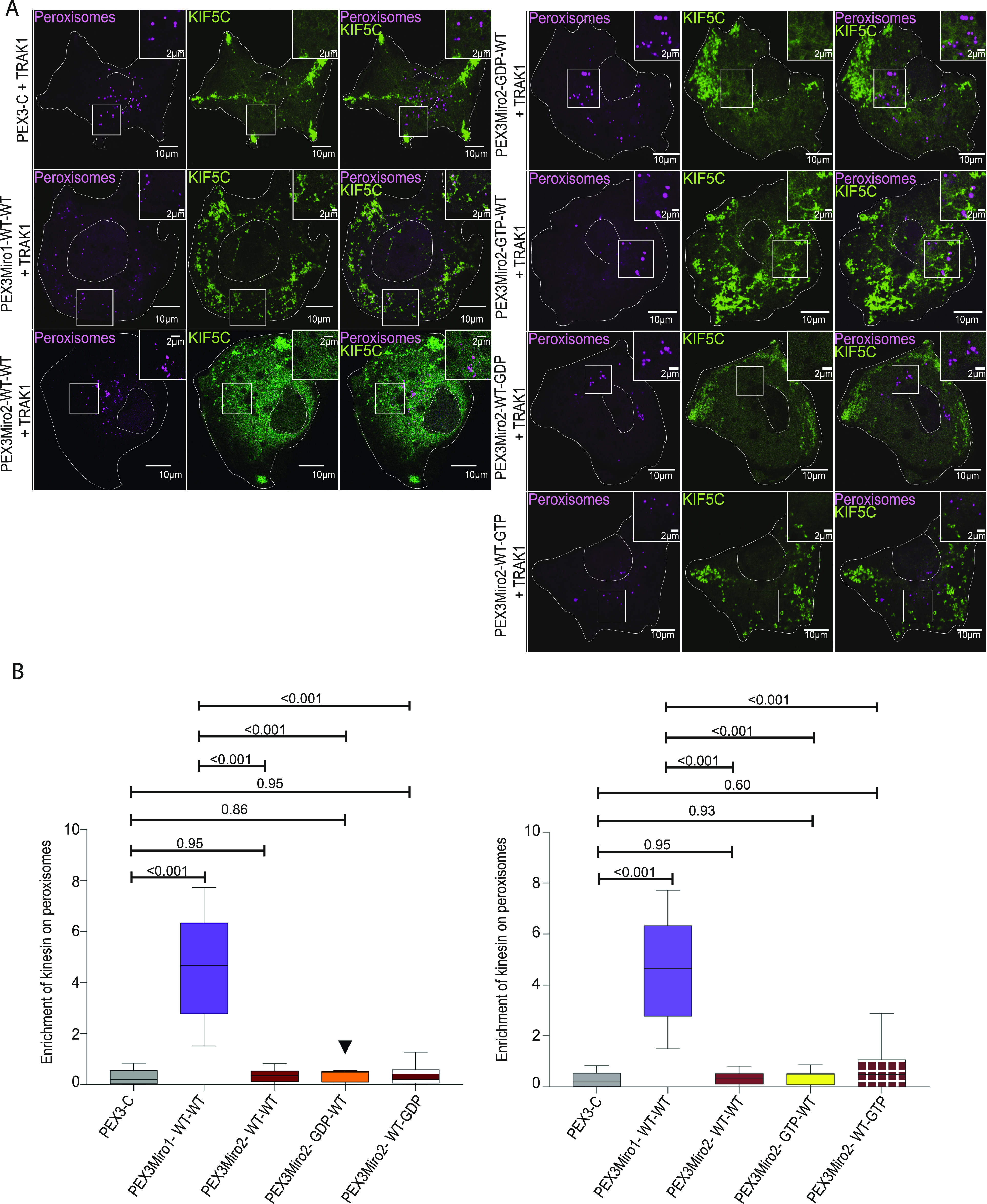
PEX3Miro2 does not recruit KIF5C to peroxisomes. **(A)** Expression in COS-7 cells of mTurquoise-SRL (magenta), myc-TRAK1, and mCitrine-KIF5C (green) with either PEX3-C, PEX3Miro1, PEX3Miro2, or PEX3Miro2 carrying a mutation of either the N- or C-GTPase domains: N-GDP-state T18N, N-GTP-state A13V, C-GDP-state S430N, and C-GTP-state A425V. The presence of the PEX3Miro2 constructs on peroxisomes was confirmed in each cell by imaging their RFP tags. The corner inserts show the enlargement of the boxed regions. Scale bar: 10 μm. Inset scale bar: 2 μm. **(B)** Quantification of the amount of KIF5C enriched on peroxisomes carrying the constructs shown in (A). For clarity, the mutations to GDP state and GTP state are plotted separately. The quantification is represented as “box-and-whisker” plots with the median value indicated. Outliers have been removed from this data set using the ROUT method (Q = 1%) and are not included in statistical calculations. Indicated *P*-values were determined by one-way ANOVA with Dunnett’s T3 correction for multiple comparisons. N = 15 cells over three biological replicates.

**Figure S7.**
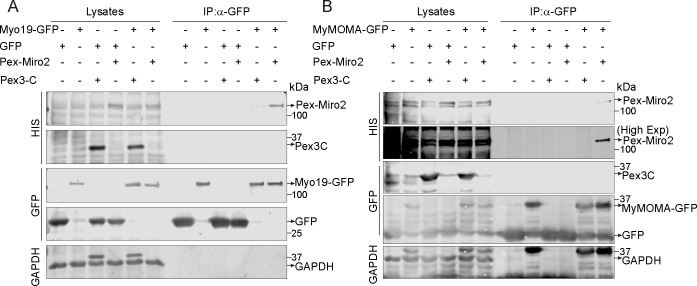
PEX3Miro2 interacts with Myo19 and its MyMOMA domain. **(A)** PEX3-C or PEX3Miro2 was expressed in HEK293T cells together with GFP or Myo19-GFP and co-immunoprecipitated using anti-GFP. Western blots were stained using anti-His to recognize the 6×His tag in the PEX3-C and PEX3Miro2 constructs, with anti-GFP to recognize Myo19, and with anti-GAPDH. **(B)** PEX3-C or PEX3Miro2 was expressed in HEK293T cells together with GFP or MyMOMA-GFP and immunoprecipitated and analyzed as above. To better detect PEXMiro2 co-IP, a longer exposure is also shown. The data are representative of three biological replicates.

Mammalian Miro1 and Miro2 are 60% identical (Fransson et al, 2003, 2006). The extent to which their functions differ remains unclear, and the role of Miro2 in mitochondrial motility has not received as much attention. The PEX3Miro approach afforded an opportunity to compare Miro1 and Miro2. We created a PEX3TM-6×His-mRFP-Miro2 construct (PEX3Miro2) equivalent to PEX3Miro1 to ask whether this peroxisomal Miro2 would interact with TRAK and kinesin. We expressed PEX3-C, PEX3Miro1, or PEX3Miro2 together with mTurquoise-SRL and mCitrine-KIF5C and with or without myc-TRAK1. PEX3Miro2 was correctly targeted to peroxisomes (Fig S6A); however, it was not able to recruit KIF5C to peroxisomes whether or not myc-TRAK1 was co-expressed (Fig 8). PEX3Miro1 co-expressed with myc-TRAK1 and assayed in parallel, recruited KIF5C as expected (Fig 8). PEX3Miro2 also failed to recruit mCitrine-TRAK1 or mCitrine-TRAK2 to peroxisomes (Fig S6B and C). This failure was not due to gross misfolding of PEX3Miro2; it coprecipitated appropriately with Myo19 and with the MyMOMA domain of Myo19 (Fig S7), known binding partners for Miro2 (Oeding et al, 2018; Bocanegra et al, 2020).

To determine whether the reason that PEX3Miro2 behaved so differently from PEX3Miro1 was due to the state of the GTPase domains it assumed when expressed in COS7 cells, we introduced mutations into the N- and C-GTPase domains of PEX3Miro2 that would create the GTP and GDP states for each domain. These constructs were co-expressed with mTurquoise-SRL and myc-TRAK1 and mCitrine-KIF5C. None of the GTPase mutations in PEX3Miro2 enabled the recruitment of the motor–adaptor complex to peroxisomes (Figs 8A and B and S6B and C). The unexpected difference in the behavior of Miro1 and Miro2 in this assay is thus unrelated to either the choice of TRAK isoform as a binding partner or the state of the GTPase domains.

### PEX3Miro1 can cause the redistribution of peroxisomes in hippocampal neurons

**Figure 9.**
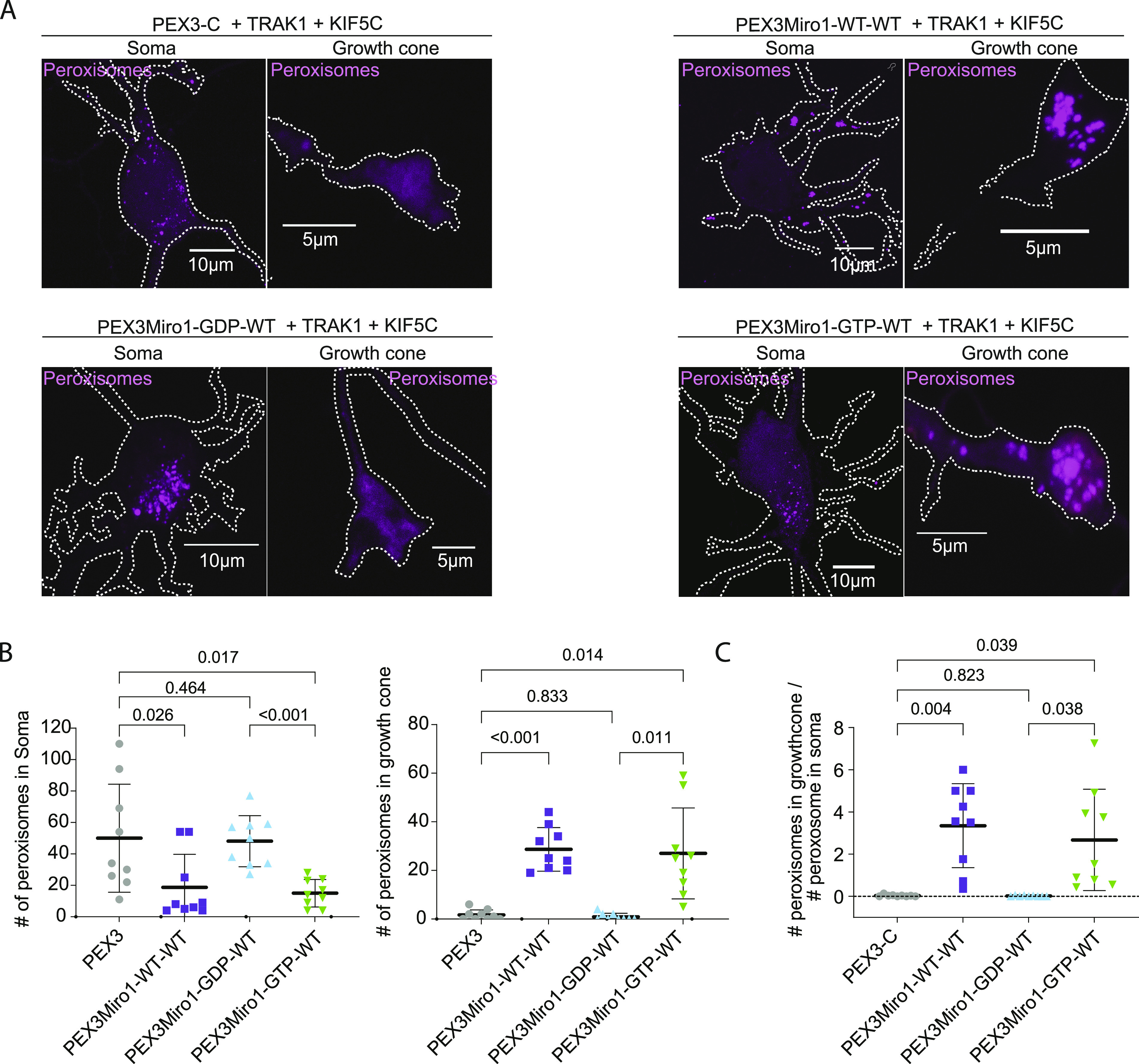
PEX3Miro1-dependent redistribution of peroxisomes in hippocampal neurons is regulated by the N-GTPase. **(A)** Expression of mTurquoise-SRL peroxisomal marker (magenta) with either PEX3 control, wild-type PEX3Miro1, or PEX3Miro1 N-GTPase mutants together with myc-TRAK1 (signal not shown) and mCitrine-KIF5C (signal not shown) in soma and growth cone of rat hippocampal neurons. Cells were fixed at DIV5. Scale bar: for soma panel, 10 μm; and for growth cone panel, 5 μm. **(B)** Quantification of the distribution of peroxisomes from neurons as in (A). For each neuron, peroxisomes within a single representative growth cone or soma were counted. All data points are plotted, N = 9 neurons from three independent experiments. For each data set, the line indicates mean, and the whisker indicates SD. The indicated *P*-values were obtained by one-way ANOVA with Dunnett’s T3 correction for multiple comparisons. **(C)** Quantification of the distribution of peroxisomes from neurons as in (A). For each neuron, peroxisomes within a single representative growth cone were counted and then expressed as a ratio relative to the number of peroxisomes in the soma of that neuron. All data points are plotted, N = 9 neurons from three independent experiments. The indicated *P*-values were obtained by one-way ANOVA with Dunnett’s T3 correction for multiple comparisons.

**Figure S8.**
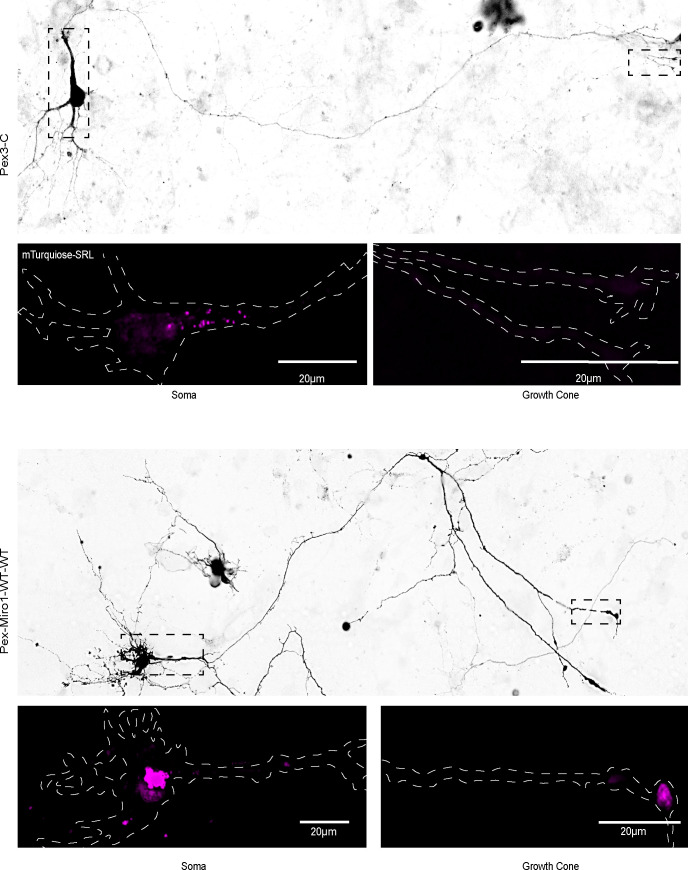
Examples of neurons from which the somatic and growth cone levels of peroxisomes were quantified. Neurons were transfected as in Fig 9, that is, with a PEX3-C or a PEX3Miro1 construct, along with mTurquoise-SRL, myc-TRAK1, and mCitrine-KIF5. The mTurquoise-SRL marker was used to visualize the distribution of peroxisomes throughout the neurons and enabled the peroxisomal counts in the soma and growth cone in Fig 9. The mCitrine-KIF5C signal was used to get a low-resolution image of the entire cell. Representative neurons expressing PEX3-C and PEX3Miro1-WT-WT are shown. Upper panels show a full neuron. The soma and growth cone whose peroxisomal signals appear below are marked (dotted lines). Scale bar: 20 μm.

**Figure S9.**
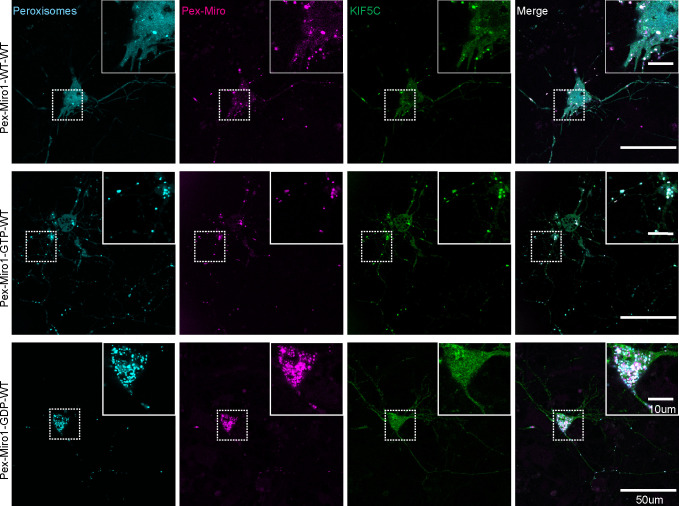
Recruitment of KIF5C to neuronal peroxisomes depends on the N-GTPase. Representative images of neurons transfected with the indicated form of PEX3Miro1, myc-TRAK1, mCitrine-KIF5C, and mTurquoise-SRL (peroxisomal marker). These neurons were among those whose peroxisomal distribution was quantified in Fig 9, and consistent with the redistribution of peroxisomes in that figure, the WT and GTP-locked forms of the N-GTPase recruited KIF5C to peroxisomes, but the GDP-bound form did not. Scale bar: 50 μm. Inset scale bar: 10 μm.

The behavior of PEXMiro1 in COS-7 cells suggested that it might also be able to alter the motility and distribution of peroxisomes in neurons and that this effect would depend on the state of the N-GTPase domain. We therefore co-expressed PEX3Miro1 and PEX3Miro1 N-GTPase mutants along with mTurquoise-SRL, myc-TRAK1, and mCitrine-KIF5C in rat hippocampal neurons. Neurons were transfected on DIV3 and fixed on DIV5 (Figs 9 and S8). To quantify their distribution, we counted the number of peroxisomes in the soma and in distal neurites. In control neurons expressing PEX3-C, peroxisomes are present throughout the neuron but are mostly localized to the soma. When we express wild-type PEX3Miro1 along with myc-TRAK1 and mCitrine-KIF5C, peroxisomes undergo a substantial redistribution, almost entirely leaving the soma and accumulating instead in distal axons and at growth cones (Figs 9 and S8). The redistributed peroxisomes had recruited KIF5C (Fig S9), and the redistribution is consistent with the KIF5C-mediated movement of peroxisomes to the plus-ends of the axonal microtubules. The same recruitment and redistribution occurred with the N-GTPase GTP-state mutant. In contrast, upon the expression of the N-GTPase GDP-state mutant with myc-TRAK1 and mCitrine-KIF5C, KIF5C was not present on peroxisomes and the vast majority of peroxisomes accumulated in the soma, with very few in the neurites (Figs 9 and S9). Thus, the Miro1 N-GTPase can alter organelle distribution in hippocampal neurons and is likely to be a major determinant of mitochondrial behavior in both neurons and non-neuronal cells.

## Discussion

By misdirecting Miro to the surface of peroxisomes, this study has allowed us to quantify the recruitment of other components of the motor–adaptor complex to Miro and to observe the consequences of that recruitment on peroxisomal distribution. The relocalization of TRAK and the motors to peroxisomes served as a readout of the ability of the complex to assemble and thereby permitted us to independently examine the two isoforms of Miro and of TRAK in a cellular milieu and reveal differences in their ability to support the assembly of the complex. In addition, the system revealed that perturbations of the N-GTPase domain of Miro altered the assembly of the complex. These findings revealed a mechanistic basis for the previously reported influences of overexpressed Miro bearing GTPase-domain mutations on mitochondrial distribution (Fransson et al, 2003, 2006; MacAskill et al, 2009a). The N-GTPase domain is a crucial regulator of microtubule-based motility, much as Miro’s EF hands were previously shown to mediate the Ca^2+^-dependent arrest of that motility (Saotome et al, 2008; Macaskill et al, 2009b; Wang & Schwarz, 2009).

By redirecting Miro constructs to peroxisomes, we avoided altering mitochondrial health or motility; mitochondria retained their endogenous Miro proteins. Although our Miro constructs, particularly if expressed at high levels, could also be found on mitochondrial membranes despite the replacement of their mitochondrial transmembrane domains with a peroxisome-targeting sequence, by restricting our analysis to peroxisomes, we could study the influence of isoforms and mutations without the confounding presence of endogenous motor–adaptor proteins on mitochondria. The “spillover” of highly expressed PEX3Miro constructs onto mitochondria was not a consequence of the Miro domains; overexpressed PEX3 alone also resided on both mitochondria and peroxisomes and to the same extent as PEX3Miro (Fig S1). One drawback of this “spillover” onto mitochondria, however, was that the PEX3Miro system was not suitable for biochemical analysis of complex assembly because too much of the protein was present on mitochondria and likely co-assembled there with endogenous Miro. We therefore restricted our analysis to the co-localization of components with the PEX3Miro-bearing peroxisomes where the ability to recruit either TRAK or a motor was strictly dependent on the nature of the PEX3Miro construct.

Both TRAK isoforms and KIF5C co-localized with peroxisomes carrying the RFP-tagged PEX3Miro1 construct, and this was accompanied by a shift in the peroxisomal localization to the periphery of the cell. We never saw either TRAK or KIF5C on peroxisomes when the PEX3 transmembrane domain without Miro1 (the PEX3-C control) was expressed, nor did PEX3-C cause the same redistribution of peroxisomes within the cell (Fig 1). Thus, although several variants of Miro1 have been reported to be expressed on peroxisomes (Costello et al, 2017; Okumoto et al, 2018; Covill-Cooke et al, 2020), if they were present on peroxisomes in our COS-7 cells, they were at too low levels to influence our assays. The co-localization data and changes in distribution were solely due to the PEX3Miro1 construct, and this was borne out by subsequent studies in which mutations of PEX3Miro1 prevented the recruitment of both TRAKs and motors. Recently, a PEX26-Miro1 fusion was reported that similarly altered peroxisomal distribution, and as in our experiments, it was found that the redistribution depended on the GTPase state of Miro1’s N-GTPase domain (Castro & Schrader, 2018). Our experiments provide a mechanistic explanation of this phenomenon by showing that motor recruitment was strictly dependent on the presence of TRAK and that TRAK could not associate with Miro1 whose N-GTPase was locked in the GDP-binding state.

The distribution of peroxisomes in COS-7 cells likely results from the combined actions of kinesin and dynein motors. By overexpressing KIF5C together with PEX3Miro1 and TRAK1, we tilted this balance in the direction of microtubule plus-ends and shifted peroxisomes toward the periphery (Fig 3). That shift was not seen when only PEX3Miro1 and TRAK1 were expressed. Expressing the P135 portion of P150, as expected, did not cause the opposite shift because P135 lacks the microtubule-binding domain and is only one component of the much larger dynein-dynactin motor.

Recently, it has been reported that two mitochondrial transmembrane proteins, metaxins 1 and 2, can also be involved in the assembly of the motor–adaptor complex (Zhao et al, 2021). We do not know whether the recruitment of TRAK, KIF5C, and P135 induced by the expression of PEX3Miro1 was independent of metaxins, whether peroxisomes contain endogenous metaxins, or whether PEX3Miro1 also caused metaxins to relocate to peroxisomes. If metaxins are needed for correct insertion and organization of Miro on mitochondria, akin to their other known functions in the mitochondrial translocation apparatus (Armstrong et al, 1997; Abdul et al, 2000), it is possible that they were not needed when Miro’s membrane association was driven by the PEX3 transmembrane domain. On the other hand, we note that PEX3Miro1 did not recruit sufficient endogenous TRAK to drive motor recruitment, which suggested that the endogenous mitochondrial Miro outcompeted PEX3Miro for TRAK. A higher affinity of mitochondrial Miro for TRAK might reflect an influence of metaxins, and further studies with PEX3Miro1 may clarify their role in the motor-adaptor complex.

The function of Miro’s GTPase domains has been of interest since Miro’s discovery and was examined chiefly by overexpressing Miro1 GTP and GDP-state mutants. N-terminal GTPase mutants altered the mitochondrial distribution and morphology in cell lines and neurons (Fransson et al, 2003, 2006; MacAskill et al, 2009a). In a *Drosophila* Miro loss-of-function mutant, the N-GTPase GDP-state mutant led to an accumulation of mitochondria in the soma of neurons and ultimately led to premature lethality (Babic et al, 2015). In Miro1/Miro2 double knockout MEFs, the Miro1 N-GTPase GTP-state mutant partially rescued mitochondrial motility, whereas the GDP-state mutant did not (Norkett et al, 2020). We offer here a mechanism behind those observations: the GDP-bound state failed to interact with either TRAK1 or TRAK2, and TRAK was absolutely required for recruiting the motors to Miro1. The N-GTPase of Miro1 also regulates the interaction between Miro1 and DISC1, CENP-F, and Myo19 (Kanfer et al, 2015; Oeding et al, 2018; Norkett et al, 2020). In these cases, as with TRAK, the Miro1 N-GTPase GDP-state mutant prevented the interactions of Miro1 that occur with wild-type Miro1 or the GTP-state mutant. Further experiments with the PEX3Miro system may determine whether the interactions of Myo19, DISC1, and CENP-F with Miro are all facilitated by the presence of TRAK or whether their dependence on the GTP state of the N-GTPase is a TRAK-independent phenomenon.

The quantitative nature of our assay revealed one aspect of the widely used P13V mutation that had not been previously appreciated: although it is predicted to lock the N-GTPase in the GTP-bound state, it was not as good as the wild-type construct at recruiting either TRAK or the motor proteins (Figs 3–5). The P13V mutation sits at the surface of the N-GTPase (Smith et al, 2020), and as such, it may play a direct role in the binding of TRAK to that domain. The P13V mutation consequently may have modestly decreased the affinity of Miro1 for TRAK, although enough TRAK and kinesin were recruited to peroxisomes to cause their redistribution and to cause the mitochondrial phenotypes reported previously.

The possibility of mutational effects that are not solely due to changing the GTP/GDP state of the protein prompted us to seek an alternative method of determining the significance of the GTPase domains. We therefore used the VopE protein from *V. cholerae*, which had previously been demonstrated with enzymatic assays to activate the Miro GTPase (Suzuki et al, 2014). As predicted by our model in which the N-GTPase controls the ability of Miro to bind TRAK, VopE expression caused TRAK to dissociate from mitochondria (Fig 6). This finding is an important confirmation of the importance of the N-GTPase as a switch that governs the assembly of the complex. Moreover, it does so by manipulating the native Miro proteins on mitochondria rather than by using peroxisomes as a surrogate organelle. VopE will likely prove to be a very useful agent for manipulating mitochondrial dynamics.

Wild-type Miro1 and the Miro1 C-GTPase mutants differed only slightly in their recruitment of kinesin to peroxisomes. In isolation, neither C-GTPase mutant differed from wild-type PEX3Miro1 in the ability to recruit TRAK1 and KIF5C to peroxisomes, nor could mutations of the C-GTPase rescue their recruitment when the N-GTPase was in the GDP state. However, when the N-GTPase carried the GTP-state mutation P13V—a state that was less efficient at recruiting TRAK1 and KIF5C—the GTP state of the C-GTPase made some differences. Heretofore, there has been little evidence for a function of the C-GTPase. The mutation of this domain has not been shown to alter interactions with Myo19, DISC1, or CENP-F (Kanfer et al, 2015), and mutations of the Miro1 C-GTPase overexpressed in COS-7 cells showed no difference in mitochondrial distribution or cell health (Fransson et al, 2003, 2006). An exception was reported in *Drosophila* where the C-GTPase GDP-state mutation decreased retrograde motility (Babic et al, 2015). More quantitative assays may detect further C-GTPase effects. From our experiments, we infer that the GTP state of the C-GTPase may promote motor complex assembly, but that it is a very subtle influence compared with the clear requirement for the GTP state of the N-GTPase.

The quantitative nature of our PEXMiro system also allowed us to examine differences between TRAK1 and TRAK2 while keeping the other components of the complex constant. The importance of the TRAKs and their *Drosophila* homolog Milton has been clear from loss-of-function phenotypes and protein interaction studies (Stowers et al, 2002; Glater et al, 2006; MacAskill et al, 2009a; Randall et al, 2013). Functional differences in the isoforms were first noted in *Drosophila* where one splicing variant, Milton-C, failed to co-immunoprecipitate with kinesin or recruit kinesin to mitochondria when expressed in COS-7 cells (Glater et al, 2006). Subsequent mammalian studies have reported functional differences between TRAK1 and TRAK2, with TRAK1 seeming to be the primary adaptor for KIF5C-driven plus-end–directed trafficking (Brickley et al, 2005; MacAskill et al, 2009a; van Spronsen et al, 2013). The difference in kinesin binding arises from a TRAK2 conformation in which a domain of TRAK2 folds back to block its kinesin-binding domain (van Spronsen et al, 2013). We, however, observed that both TRAK1 and TRAK2 could recruit kinesin to PEX3Miro1 peroxisomes but that TRAK1 was quantitatively more effective than TRAK2. This observation aligns with the differences previously found between TRAK1 and TRAK2 but also suggests that, in a cellular context, TRAK2 is not exclusively in its closed state and can also support plus-end–directed mitochondrial transport by KIF5C.

We were surprised to find that PEX3Miro2 failed to recruit either TRAK, KIF5C, or P135 to peroxisomes and that this failure could not be reversed by mutations of the GTPase domains. We do not think this is a consequence of the N-terminal tags and misfolding of the protein because a similarly tagged form of Miro2 was shown to bind Myo19 (Bocanegra et al, 2020), and we confirmed that PEX3Miro2 does as well (Fig S7). It is possible that Miro2 requires additional factors present on mitochondria that are not on peroxisomes, such as metaxins, but it is also possible that Miro2 does not mediate mitochondrial transport by microtubule-based motors. Miro2 has received less attention than Miro1, and there is less evidence for the role of Miro2 in driving long-range mitochondrial motility. Loss of Miro2 had little effect on mitochondrial motility in hippocampal neurons (Lopez-Domenech et al, 2016). The persistence of some processive mitochondrial movements in cells lacking Miro1 and the severe phenotypes of Miro1 and 2 double knockouts (Nguyen et al, 2014; Lopez-Domenech et al, 2016) has suggested some redundancy in their function. Although both overexpressed Miro1 and Miro2 localize to all mitochondria (Fransson et al, 2003), it is not known whether this is true of the endogenous proteins or whether they localize to different populations of mitochondria or different sites on mitochondria. Myo19 does interact with Miro2 to support actin-based mitochondrial transport (Oeding et al, 2018; Bocanegra et al, 2020), and Miro2 is implicated in tethering mitochondria to ER (Modi et al, 2019; White et al, 2020). Thus, the sharp difference in the behavior of PEX3Miro1 and PEX3Miro2 reported here indicates that further attention to their functional differences is needed. We note that our data disagree with those of Fransson et al (2006) who studied the overexpression of Miro and TRAK and found that TRAK coprecipitated with both Miro1 and Miro2 and did so regardless of the state of the N-GTPase. We propose that this was due to the co-assembly of the overexpressed forms of Miro with the endogenous wild-type Miro1, which in turn bound to TRAK.

GTPases are regulated by GTPase-activating proteins (GAPs) and guanine nucleotide exchange factors (GEFs). A Miro GAP and GEF would be expected in cells if the GTPase domains are behaving in vivo as regulatory switches. VopE, a protein expressed by *Vibrio cholera* type III in infected cells, behaves as a GAP for Miro. By converting the N-GTPase of both Miro1 and Miro2 to the GDP state, it alters the distribution of the host cell’s mitochondria in a manner consistent with our findings (Suzuki et al, 2014). Our data predicted that VopE would do so by causing the dissociation of TRAK from Miro and thereby from the mitochondrial surface. Indeed, by co-expressing VopE with mCitrine-TRAK1 we demonstrated that dissociation (Fig 7). The activity of a GAP from a pathogen begs the question as to what endogenous cellular proteins function as Miro GAPs and when they are called upon to disassemble the mitochondrial motor–adaptor complex. No such endogenous Miro GAPs are known at present. Vimar, however, the *Drosophila* homolog of RAP1GDS1, may be a potential Miro GEF; vimar mutations alter mitochondrial morphology in *Drosophila*, and RAP1GDS1 knockdown rescues damaged mitochondrial phenotypes in mammalian cells (Ding et al, 2016). RAP1GDS1 coprecipitates with Miro1, but it has not been shown to alter the GTP/GDP state of Miro as predicted for a GEF. GBF1, a GEF for Arf1 GTPase (Gillingham & Munro, 2007), also influences mitochondrial morphology and position (Walch et al, 2018) and is a candidate for a cellular Miro GEF. Changes in mitochondrial morphology and distribution, however, can reflect many factors beyond the activity of the microtubule-based motors, including the fusion and fission apparatuses and mitochondrial contacts with other organelles. The PEX3Miro system may be valuable for identifying cellular GEFs and GAPs that control Miro1 and thereby control mitochondrial movement.

From our imaging of peroxisomes in neurons and COS-7 cells, it is clear that the state of the N-GTPase can have a profound effect on organelle distribution. These changes in distribution are consonant with a model in which the N-GTPase is a switch that controls TRAK, kinesin, and P135 assembly into a Miro1-based motor–adaptor complex. We do not know when in a neuron the N-GTPase is in the GDP state. In neurons expressing labeled kinesin, all the axonal mitochondria, including those that are stationary, have kinesin on them (Wang & Schwarz, 2009) and some signals that trigger mitochondrial arrest, including elevated Ca^2+^ or glucose, do not cause kinesin to fall off mitochondria (Wang & Schwarz, 2009; Basu et al, 2021); these signals cannot be acting through the N-GTPase. Motors are shed from mitochondria, however, during metaphase in mitotic cells (Chung et al, 2016), and it is possible that this is accompanied or mediated by a change in the GTP state of Miro. Understanding the mechanism by which the GTPase switch influences the complex, as revealed by the present studies, should facilitate elucidating when and how cells use this switch to control mitochondrial behavior.

## Materials and Methods

### Primers

Sequences of all primers used in this study are presented in Table S1.

Table S1 Table of primers used for all plasmids made for this study.

### Plasmid constructs

#### Previously published constructs

The following previously published DNA constructs were used in this study: pmTurquoise2-Peroxi was a gift from Dorus Gadella (University of Amsterdam [Addgene plasmid # 36203; http://n2t.net/addgene:36203; RRID:Addgene_36203] [Goedhart et al, 2012]); pmTurquoise2-Mito was a gift from Dorus Gadella (University of Amsterdam [Addgene plasmid # 36208; http://n2t.net/addgene:36208; RRID:Addgene_36208] [Goedhart et al, 2012]); mCitrine-Peroxisomes-2 was a gift from Michael Davidson (National MagLab [Addgene plasmid # 54672; http://n2t.net/addgene:54672; RRID:Addgene_54672]); and mCitrine-C1 was a gift from Robert Campbell & Michael Davidson & Oliver Griesbeck & Roger Tsien (Howard Hughes Medical Institute, University of California, San Diego, and National MagLab [Addgene plasmid # 54587; http://n2t.net/addgene:54587; RRID:Addgene_54587] [Griesbeck et al, 2001]). mycMiro1-V13, mycMiro1-V427, mycMiro1-N18, and mycMiro1-N432 were kindly provided by Dr. P Aspenstrom (Karolinska Institute). The construction of Myc-TRAK1 and PEX3-6×His-mRFP-Miro1 in our laboratory was previously described (Pekkurnaz et al, 2014; Basu et al, 2021).

#### Purchased constructs

mCitrine-P135 was subcloned by VectorBuilder from mCitrine-P150^Glued^.

#### Constructs cloned in this study

(1) To make the *PEX3-6×His-mRFP* construct (PEX3-C), the *PEX3-6×His-mRFP-Miro1* construct (PEX3Miro1) described in Basu et al (2021) was linearized by PCR using primers *KP14* and *KP15* to amplify the *PEX3-6×His-mRFP* segment of the plasmid and the plasmid backbone but omit Miro1. The PCR product was ligated using the KLD reagent from the Q5 site-directed mutagenesis kit from NEB. This construct was used as a control throughout all co-localization experiments.
(2) To make the *Myc-TRAK2* construct, the *Myc-TRAK1* construct described in Pekkurnaz et al (2014) was linearized by PCR with primers *KP51* and *KP52*, thereby excluding the *TRAK1* sequence from a vector still containing the myc-sequence. A *TRAK2* cassette was amplified from a *TRAK2* cDNA (BC048093; transOMIC Technologies Inc.) using *KP49* and *KP50* primers that had appropriate overhangs to the myc-vector for recircularization by the Gibson assembly. This construct was used to overexpress Myc-TRAK2 in kinesin co-localization experiments.
(3) *mCitrine-KIF5C-YFP* was derived from the *mCitrine-KHC-eCFP* construct generously gifted by Kristen Verhey (University of Michigan [Cai et al, 2007]). The eCFP tag was substituted with a YFP tag.
(4) To make the *mCitrine-TRAK1-YFP* and *mCitrine-TRAK2-YFP* constructs, the *mCitrine-KIF5C-YFP* was linearized by PCR with primers *KP61* and *KP62* for TRAK1 and *KP65* and *KP66* for TRAK2. These primers were designed to omit the *KIF5C* from the backbone while retaining the *mCitrine-YFP* backbone sequence. The *TRAK1* and *TRAK2* cassettes were amplified from the *myc-TRAK1* construct (Pekkurnaz et al, 2014) and the *myc-TRAK2* construct described in this study using *KP63* and *KP64* for *TRAK1* and primers *KP67* and *KP68*. The primers had appropriate overhangs to the *mCitrine-YFP* vector for recircularization by Gibson assembly after incorporating the *TRAK1* and the *TRAK2* cassettes downstream of mCitrine. These constructs were used in all *mCitrine-TRAK1* or *mCitrine-TRAK2* co-localization experiments.
(5) The *PEX3-6×His-mRFP-Miro2* construct was made by amplifying the first 592–amino acid residues from human Miro2 as cloned in Stowers et al (2002), Fransson et al (2003, 2006), Glater et al (2006), and Brickley and Stephenson (2011) with primers *pex-miro2_ForPrimer* and *pex-miro2_RevPrimer*. The backbone containing *PEX3-6×His-mRFP* was amplified from the *PEX3-6×His-mRFP-Miro1* using primers *pexBackbone_ForPrimer* and *pexBackbone_RevPrimer*. The two PCR fragments were then annealed by Gibson assembly. All the mutations were made by site-directed mutagenesis carried out on the PEX3Miro1 and PEX3Miro2 constructs.
(6) To generate the *mTurquoise-KIF5C* construct, the previously described *mCitrine-KIF5C-YFP* plasmid, was used to amplify *KIF5C* using *KP69* and *KP70* primers that had appropriate overhangs for the *mTurquoise* vector. The *mTurquoise* plasmid vector was amplified from *pmTurquoise2-Peroxi* (Addgene plasmid # 36203[Goedhart et al, 2012]) using *KP71* and *KP72* primers. The linearized *mTurquoise* vector was recircularized after incorporating the *KIF5C* cassette at the C-terminus of mTurquoise by Gibson assembly. These constructs were used to overexpress mTurquoise-KIF5C in co-localization experiments.
(7) All PEX3Miro1 GTPase mutants were made using the Q5 Site-Directed Mutagenesis Kit from New England Biolabs. The T18N mutation was made using *KP24* and *KP25*. The P13V mutation was made using *KP26* and *KP27*. The K427N mutation was made using *KP146* and *KP147*. The S432N mutation was made using *KP31* and *KP32*. For mutations where both the N- and C-GTPases were mutated, we used the N-GTPase mutant as a template for introducing the C-GTPase mutation with the primers mentioned above.
(8) All PEX3Miro2 GTPase mutants were made using the Q5 Site-Directed Mutagenesis Kit from New England Biolabs. The T18N mutation was made using *KP78* and *KP79*. The A13V mutation was made using *KP76* and *KP77*. The A425V mutation was made using *KP82* and *KP83*. The S430N mutation was made using *KP102* and *KP81*.
(9) To make mCitrine-P150^Glued^, we used the *mCitrine-C1* cloning vector (Griesbeck et al, 2001) and digested the vector with HindIII and Kpn1 enzymes. The P150^Glued^ cassette was amplified from the *pEGFPC2-P150*^*Glued*^ construct provided by Dr. EL Holzbaur (University of Pennsylvania) using primers KP112 and KP113 with overhangs with HindIII and Kpn1 enzyme cut sites. The amplified cassette was digested by HindIII and Kpn1 enzymes, and the *mCitrine-C1* vector and *P150* insert were ligated using T4 DNA ligase (New England Biolabs).
(10) To generate the *VopE* construct, a codon-optimized sequence of the gene (Suzuki et al, 2014) was synthesized that was flanked by EcoRI and KpnI restriction sites. This sequence was inserted into the *mEGFP-N1* backbone (Addgene plasmid # 54767) and thereby introduced a frameshift to prevent the expression of the downstream *mEGFP*.

### Antibodies used for Western blotting

For Western blots, the following primary antibodies were used at the stated dilutions: anti-human TRAK1 at 1:2,000 (HPA005853; Sigma-Aldrich), anti-myc at 1:5,000 (05-724; EMD Millipore), anti-RFP at 1:1,000 (SAB2702214; Sigma-Aldrich), anti-GFP at 1:1,000 (GFP-1020; Aves Labs), anti-GAPDH at 1:1,000 (2118S; Cell Signaling Technology), and anti-6×His at 1:1,000 (MA1-21315; Thermo Fisher Scientific). For fluorescence detection (used for all quantitative blots), 680RD Donkey anti-Rabbit and 800CW Donkey anti-Mouse antibodies were used at 1:5,000 (LI-COR Biosciences) and all blots were scanned by the Odyssey CLx Imaging System.

### Cell cultures and transfections

HEK293T and COS-7 cells were cultured in DMEM supplemented with L-glutamine, penicillin/streptomycin (Life Technologies), and 10% FBS (Atlanta Premium). Plasmid DNA transfections in HEK293T cells were performed with calcium phosphate (Kingston et al, 2003). Plasmid DNA transfections in COS-7 cells were performed with PolyJet DNA (SignaGen Laboratories) using the manufacturer’s guidelines. These cell lines were generally transfected 18–24 h after plating and fixed 2 d later.

### Hippocampal neurons

Hippocampal neurons were dissected and dissociated from E18 rat (Charles River, Sprague Dawley) embryos as previously described (Nie & Sahin, 2012) and plated at 5–7 × 10^4^/cm^2^ density on glass bottom dishes (D35-20-1.5-N; Cellvis) coated with 20 μg/ml poly-L-lysine (Sigma-Aldrich) and 4 μg/ml laminin (Life Technologies) and maintained in neurobasal medium supplemented with B27 (Life Technologies), L-glutamine, and penicillin/streptomycin, unless specified otherwise. Hippocampal neurons were transfected on DIV3 using Lipofectamine 2000 (11668-019; Life Technologies) and fixed and imaged 2–3 d later. All rat experimental procedures were performed in compliance with the Boston Children’s Hospital Institutional Animal Care and Use Committee (protocol# 15-09-3039R) and the Charles River Animal Welfare and the Humane Treatment of Animals Policy.

### Immunoprecipitation

For immunoprecipitation of myc-TRAK1 and mCitrine-KIF5C with PEX3-C and PEX3Miro1, HEK293T cells were plated at 5.5 × 10^5^ cells/well density in a six-well plate and transfected the next day. 2 d after transfection, cells were washed once with ice-cold 1× PBS (NaCl: 137 mM, KCl: 2.7 mM, Na_2_HPO_4_: 10 mM, and KH_2_PO_4_: 1.8 mM) and lysed in 600 μl PierceTM IP lysis buffer (Cat. no.: 87787; Thermo Fisher Scientific) and protease inhibitor cocktail set III (539134-1SET; EMD Millipore) at 1:500 dilution. Lysates were centrifuged for 10 min at 13,000*g* at 4°C, and the clarified supernatants were collected. For immunoprecipitation of PEX3-6×His-mRFP-Miro, anti-6×His antibody was incubated for 1 h at 4°C with 500 μl of the clarified supernatants of whole-cell lysates and then for one more hour with Dynabeads Protein G beads (Cat. no.: 10003D; Thermo Fisher Scientific). The beads had been pre-blocked in 0.2% TBST and washed three times with lysis buffer. After magnetic separation, beads were washed five times in lysis buffer and resuspended in 2×Laemmli buffer. 50% of this fraction was then separated by SDS–PAGE and transferred to nitrocellulose membranes. The membranes were pre-blocked overnight with 3% bovine serum albumin (wt/vol) in 1× TBS with 0.1% (wt/vol) Tween-20 (TBS: 20 mM Tris, NaCl: 150 mM), followed by overnight incubation at 4°C with primary antibodies in the blocking buffer. The blot was then washed three times with TBST and blotted with secondary antibodies for 1 h at room temperature, before ECL imaging or being scanned by the Odyssey CLx Imaging System.

For immunoprecipitation of PEX3Miro2 with Myo19 or the MyMOMA fragment, HEK293T cells were plated at 4 × 10^5^ cells/well density in a six-well plate, and the next day, two wells were transfected per experimental condition. 24 h after transfection, cells were washed twice with ice-cold 1× PBS (NaCl: 137 mM, KCl: 2.7 mM, Na_2_HPO_4_: 10 mM, and KH_2_PO_4_: 1.8 mM) and lysed in 300 μl of lysis buffer per condition (10 mM Tris–HCl, pH 7.4, 150 mM NaCl, 1.5 mM MgCl_2_, 5 m EDTA, 1% Triton, 10% glycerol, 1 mM NaF, and 50 mM Na_2_H_2_P_2_O_7_) and protease inhibitor cocktail set III (539134-1SET; EMD Millipore) at 1:500 dilution. Lysates were centrifuged for 20 min at 13,000*g* at 4°C, the clarified supernatants were collected, and 24 μl of them was resuspended with 8 μl of 4× Laemmli buffer. For immunoprecipitation of Myo19-GFP and MyMOMA-GFP, 4 μl of anti-GFP-Trap agarose (ChromoTek) was incubated o/n at 4°C with 300 μl of the clarified supernatants after the addition of 20 μl of 10% (wt/vol) BSA. Beads were then washed five times in wash buffer (10 mM Tris–HCl, pH 7.5, 500 mM NaCl, 1% Triton, 0.5% NP-40, 1 mM MgCl_2_, 1 mM EDTA, and 0.5 mM EGTA) and resuspended in 4×Laemmli buffer. Lysates and immunoprecipitates were then separated by SDS–PAGE and transferred to nitrocellulose membranes. The membranes were pre-blocked overnight with 5% BSA (wt/vol) in 1× TBS with 0.1% (wt/vol) Tween-20 (TBS: 20 mM Tris, NaCl: 150 mM), followed by overnight incubation at 4°C with primary antibodies in the blocking buffer. The blot was then washed three times with TBST and blotted with secondary antibodies for 1 h at room temperature, before ECL imaging or being scanned by the Odyssey CLx Imaging System.

### Imaging acquisition and quantification

For imaging co-localization of TRAK1, TRAK2, P135, and KIF5C on PEX3Miro1 peroxisomes, COS-7 cells were grown on glass bottom dishes (FD35-100; World Precision Instruments). Cells were fixed and imaged at room temperature. For imaging the distribution of PEX3Miro1 peroxisomes in rat hippocampal neurons, primary neurons were isolated as described and grown on glass bottom dishes (FD35-100; World Precision Instruments). Neurons were fixed 48 h after transfection and imaged at room temperature. COS-7 cells and neurons were imaged on a Leica SP8 laser scanning confocal microscope. Images were acquired using a 60× objective. A white light laser and argon laser were used at 70% laser intensity, and line scanning with three scans per line was used to acquire all images. The percentage of laser intensity used for the 458 spectra to capture mTurquoise markers was 5% in all experiments. The percentage of laser intensity used for the 515 spectra to capture mCitrine-tagged constructs was 10%. The percentage of laser intensity used for the 554 spectra to capture all mRFP-tagged constructs was 10%. The gain was set at 100 for imaging mCitrine-TRAK1, mCitrine-TRAK2, mCitrine-KIF5C, PEX3-C, and PEX3Miro for all COS-7 and hippocampal neuron images. Only cells with visible PEX3-C or PEX3Miro signals on peroxisomes at 100 gain with these laser settings were imaged to minimize differences in expression. Images were acquired using z-stacks with 0.3-μm slices for COS-7 cells and 0.2-μm slices for neurons, ranging from the top to the bottom of the cell or neuronal soma. For imaging co-localization of TRAK1 on mitochondria, COS-7 cells were grown as above and cells were live-imaged at 37°C and 5% CO_2_ on a Nikon Ti Eclipse inverted microscope. Images were acquired using z-stacks with 0.3-μm slices with a 60× objective.

Images for Fig S8 were obtained through the Leica DMi8 Thunder microscope system with an sCMOS camera having a pixel size of 6.5 μM (DFC 9000) and a 40× 0.8NA objective. Following image acquisition, images were processed through the small volume computational clearing pipeline, proprietary to the thunder imaging system. Each neuron was imaged as a series of areas later stitched together to visualize the entire neuron. In addition to imaging in single channels, neurons were also illuminated simultaneously for CFP (mTurquoise-SRL) and YFP (mCitrine-KIF5C-YFP) fluorescence to obtain images that represent the whole neuron (cell fill).

### Quantifications of organelle distribution in COS7 cells

The quantification of peroxisomal distribution was done as described in Basu et al (2021) using the DoveSonoPro software (https://github.com/ThomasSchwarzLab). The DoveSonoPro software allows for the cell outline and cell center to be selected by the user. Image file names were blinded for quantification.

### Co-localization quantification

The co-localization of TRAK1/2, KIF5C, and P135 with PEX3-C or PEX3Miro peroxisomes was quantified using a custom FIJI macro. Images of cells were acquired using z-stacks with 0.3-μm thickness, and z-slice images were acquired from the top to the bottom of cells to account for all peroxisomes. This custom macro creates a mask of the peroxisomal marker channel (mTurquoise-SRL or mCitrine-PTS1) in each z-slice and uses the peroxisomal mask to analyze only the pixels in the image where the peroxisomal marker co-localizes with the RFP-tagged PEX3-C or PEX3Miro construct. The PEX3-C and PEX3Miro positive peroxisomes are then analyzed for co-localization of KIF5C, TRAK1, TRAK2, and P135. The final quantification is the average of the raw enrichment of KIF5C, TRAK1, TRAK2, or P135 across z-slices on PEX3-C or PEX3Miro1 positive peroxisomes. Because the expression of the PEX3 control or PEX3Miro1 construct varies slightly from cell to cell, we measured the intensity of the PEX3-C and PEX3Miro construct that is localized to peroxisomes. This macro can control for any PEX3-C or PEX3Miro1 expression-dependent differences in co-localization by normalizing the raw amount of KIF5C, TRAK1, TRAK2, or P135 to the raw intensity of PEX3-C or PEX3Miro expression. We controlled all experiments by ruling out any expression-dependent differences. We then control for potential error in co-localization quantification by taking into consideration the partial cytosolic localization of any overexpressed mCitrine-KIF5C, TRAK1, TRAK2, or P135. To do this, we subtract the whole-cell intensity of the mCitrine signal that exists outside of the peroxisomal mask from the specific signal intensity that overlaps with the peroxisomal mask. This background fluorescence subtraction allows for the quantification of the specific amount of signal on PEX3-C or PEX3Miro peroxisomes without confounding results with the presence of the cytosolic localization of the overexpressed constructs.

The co-localization of mCitrine-TRAK1-YFP on mitochondria was quantified using a custom FIJI macro based on that used for peroxisomes. The macro creates a mask of the mitochondrial marker channel (mtDsRed) in each z-slice and uses the TRAK1-YFP mask to analyze only the pixels in the image where the YFP marker co-localizes with the mtDsRed. The final quantification is the average of the raw enrichment of TRAK across z-slices on mtDsRed relative to the cytosol.

### Statistical analysis

Statistical analysis was performed with GraphPad Prism v7.0. A two-tailed unpaired *t* test with Welch’s correction or a one-way ANOVA with Dunnett’s T3 correction was used to determine significant differences between populations in all peroxisomal co-localization experiment quantifications. *P*-values appear in all the figures. All co-localization quantifications for peroxisomes are depicted as Tukey’s boxplots. Mitochondrial co-localization was analyzed similarly, but GraphPad Prism v8.0 and the Mann–Whitney test were used to determine the *P*-value.

## Supplementary Material

Reviewer comments
