# Supplementary material for: Miro GTPase domains regulate the assembly of the mitochondrial motor–adaptor complex
Source: Life Sci Alliance. 2022 Oct 27;6(1):e202201406. doi: 10.26508/lsa.202201406 (PMC9615026; doi:10.26508/lsa.202201406)
Supplement: Supplementary file 1 [file LSA-2022-01406_TableS1.docx]

***Table S1***Table of primers used for all plasmids made for this study

| **Primer Name** | **Sequence** | **Notes** |
| --- | --- | --- |
| KP14 (forward) | ACTAGTTATTAAGAATTCATTGATCATAATCAGCCA | Primers to amplify the PEX3-6xhis-mRFP cassette from the PEX3-6xhis-mRFP-Miro1 plasmid. |
| KP15 (reverse) | AGAATTCGAAGCTTGAGCTCGAGATCTGAG |  |
|  | | |
| KP24 (forward) | GAAGAACTCACTGATTATGTC | Primers used for site directed mutagenesis to introduce the T18N Miro1 N-GTPase mutation in PEX3Miro1 |
| KP25 (reverse) | CCAACTCTAGGTTCTCCCACC |  |
|  | | |
| KP26 (forward) | GAAGTTAGAGTTGGGAAGACA | Primers used for site directed mutagenesis to introduce the P13V Miro1 N-GTPase mutation in PEX3Miro1 |
| KP27 (reverse) | TCCCACCAGCAGGATCCGCAC |  |
|  | | |
| KP31 (reverse) | GTTTTTCACTCCAATTACATT | Primers used for site directed mutagenesis to introduce the S432N Miro1 C-GTPase mutation in PEX3Miro1 |
| KP32 (forward) | TGTGGGAAAAATGGAGTTCTTCAG |  |
|  | | |
| KP49 (forward) | CGGGCGGATCCAATGAGTCAATCCCAGAATGCA | Primers used to amplify the TRAK2 cDNA with binding regions for the myc-tagged backbone |
| KP50 (reverse) | CCCCCTCGAGACTCAGTCCTCCTTCAGGACACC |  |
| KP51 (forward | AGGAGGACTGAGTCTCGAGGGGGGGCC | Primers used to linearize the myc-tagged backbone from the myc-TRAK1 plasmid. Primers have binding regions for the TRAK2 cDNA cassette. |
| KP52 (reverse) | TTGACTCATTGGATCCGCCCG |  |
|  | | |
| KP61 (forward) | AACTAGCTTACGGGGAGCAGGTACTGGAGC | Primers to linearize the mCitrine-YFP backbone from the mCitrine-KIF5C-YFP plasmid. Primers have overhangs for the TRAK1 cassette. |
| KP62 (reverse) | AAAAACCAATGCTCCTGCTCCGGACTTGTA |  |
| KP63 (forward) | TCCGGAGCAGGAGCATTGGTTTTTCAATTC | Primers to amplify the TRAK1 cassette from the myc-TRAK1 plasmid with primer overhangs for the mCitrine-YFP backbone. |
| KP64 (reverse) | TCCAGTACCTGCTCCCCGTAAGCTAGTTTG |  |
|  | | |
| KP65 (forward) | CTGAAGGAGGACGGAGCAGGTACTGGAGCA | Primers to linearize the mCitrine-YFP backbone from the mCitrine-KIF5C-YFP plasmid. Primers have overhangs for the TRAK1 cassette. |
| KP66 (reverse) | CTGGGATTGACTTCCTGCTCCGGACTTGTA |  |
| KP67 (forward) | TCCGGAGCAGGAAGTCAATCCCAGAATGCA | Primers to amplify the TRAK2 cassette from the myc-TRAK2 plasmid with primer overhangs for the mCitrine-YFP backbone. |
| KP68 (reverse) | AGTACCTGCTCCGTCCTCCTTCAGGACACC |  |
|  | | |
| KP69 (forward) | GAGCTGTACAAGTCCGGAGCAGGAATGGCG | Primers to amplify the KIF5C cassette from the mCitrine-KIF5C-YFP plasmid with primer overhangs for the mTurquoise backbone. |
| KP70 (reverse) | TCCGGTGGATCCCTTCTGGTAGTGAGTGGA |  |
| KP71 (forward) | CACTACCAGAAGGGATCCACCGGATCTAGA | Primers to linearize the mTurquoise backbone from the mTurquoise-SRL plasmid. Primers have overhangs for the KIF5C cassette. |
| KP72 (reverse) | TCCTGCTCCGGACTTGTACAGCTCGTCCAT |  |
|  | | |
| KP76 (forward) | GAGGTTCAGGTGGGGAAGACG | Primers used for site directed mutagenesis to introduce the A13V Miro2 N-GTPase mutation in PEX3Miro2 |
| KP77 (reverse) | GCCCAGTAACAGGATGCGCAC |  |
|  |  |  |
| KP78 (forward) | GGGAAGAACTCGCTGATCCTG | Primers used for site directed mutagenesis to introduce the T18N Miro2 N-GTPase mutation in PEX3Miro2 |
| KP79 (reverse) | CACCTGGGCCTCGCCCAGTAA |  |
|  | | |
| KP81 (reverse) | GCCCACTCCACAGGCCCCTAC | Primers used for site directed mutagenesis to introduce the S430N Miro2 C-GTPase mutation in PEX3Miro2 |
| KP102 (forward) | AAGAACGCCTTCCTGCAGGCC |  |
|  | | |
| KP82 (forward) | GGGGTTTGTGGAGTGGGCAAG | Primers used for site directed mutagenesis to introduce the A425V Miro2 C-GTPase mutation in PEX3Miro2 |
| KP83 (reverse) | TACCACCTTGCACAGGAGGAC |  |
|  | | |
| KP112 (reverse) | GGAAGGAACCATGGATCAGTTATCTAGATCCGGTGGATCCCGGG | Primers used to amplify the P150-Glued cassette from the pEGFPC2-P150Glued plasmid gifted from E.L. Holzbaur. The Primers have overhangs with HindIII and KPN1 enzyme cut sites |
| KP113 (forward) | CCTTCCTTAAGCTTCTATGGCACAGAGCAAGAGGCAC |  |
|  | | |
| KP146 | GGAGTGAATAACTGTGGGAAA | Primers used for site directed mutagenesis to introduce the K427N Miro1 C-GTPase mutation in PEX3Miro1 |
| KP147 | AATTACATTACATCTGAACAC |  |
|  | | |
| pex-Miro2_ForPrimer | *CTCAGATCTCGAGCTCAAGCTTCGAATTCTATGAGGCGGGACGTGCGCATCCTGTTACTG* | Primers used to amplify the Miro2 cassette from the hMiro2 cloned in (Fransson et al., 2003). The Primers have overhangs for the PEX3-6xhis-mRFP backbone Underlined regions show the binding regions of primers to Miro2 |
| pex-miro2_RevPrimer | *AGATCCGGTGGATCCCGGGCCCGCGGTACCCCGGAGCCAGAAGGAAGAGGGATGCAG* |  |
| pexBackbone_ForPrimer | *GTACCGCGGGCCCGGGAT* | Primers used to amplify the PEX3-6xhis-mRFP backbone. |
| pexBackbone_RevPrimer | *AATTCGAAGCTTGAGCTCGAGATCTGAGTC* |  |
